# Genetic control of pod morphological traits and pod edibility in a common bean RIL population

**DOI:** 10.1007/s00122-023-04516-6

**Published:** 2023-12-13

**Authors:** Carmen García-Fernández, Maria Jurado, Ana Campa, Elena Bitocchi, Roberto Papa, Juan Jose Ferreira

**Affiliations:** 1 Plant Genetic Group, Regional Service for Agrofood Research and Development (SERIDA), 33300 Villaviciosa, Asturias Spain; 2 Department of Agricultural, Food, and Environmental Sciences, Marche Polytechnic University, Via Brecce Bianche, 60131 Ancona, Italy

## Abstract

**Key message:**

QTL mapping, association analysis, and colocation study with previously reported QTL revealed three main regions controlling pod morphological traits and two loci for edible pod characteristics on the common bean chromosomes Pv01 and Pv06.

**Abstract:**

Bean pod phenotype is a complex characteristic defined by the combination of different traits that determine the potential use of a genotype as a snap bean. In this study, the TUM RIL population derived from a cross between ‘TU’ (dry) and ‘Musica’ (snap) was used to investigate the genetic control of pod phenotype. The character was dissected into pod morphological traits (PMTs) and edible pod characteristics (EPC). The results revealed 35 QTL for PMTs located on seven chromosomes, suggesting a strong QTL colocation on chromosomes Pv01 and Pv06. Some QTL were colocated with previously reported QTL, leading to the mapping of 15 consensus regions associated with bean PMTs. Analysis of EPC of cooked beans revealed that two major loci with epistatic effect, located on chromosomes Pv01 and Pv06, are involved in the genetic control of this trait. An association study using a subset of the Spanish Diversity Panel (snap vs. non-snap) detected 23 genomic regions, with three regions being mapped at a position similar to those of two loci identified in the TUM population. The results demonstrated the relevant roles of Pv01 and Pv06 in the modulation of bean pod phenotype. Gene ontology enrichment analysis revealed a significant overrepresentation of genes regulating the phenylpropanoid metabolic process and auxin response in regions associated with PMTs and EPC, respectively. Both biological functions converged in the lignin biosynthetic pathway, suggesting the key role of the pathway in the genetic control of bean pod phenotype.

**Supplementary Information:**

The online version contains supplementary material available at 10.1007/s00122-023-04516-6.

## Introduction

The common bean (*Phaseolus vulga*ris L.) occupies one of the top positions in the global ranking of grain legumes, with an average global production of 529 million tons over the last decade (http://www.fao.org/faostat/, accessed on 11 November 2022). In addition to its consumption as a grain legume (dry beans), some common bean genotypes, known as snap beans (also known as green beans or French green beans) have succulent immature pods with reduced insoluble fiber (< 20%) and are suitable for consumption as vegetables (Myers and Baggett 1999). Unlike dry beans, snap beans are harvested during the early developmental stage, usually when their immature pods have reached the maximum pod length, but the pod filling process is at an early-intermediate stage [beginning of R8 stage (Schoonhoven and van Pastor Corrales 1987)]. Snap beans have a high water content (~ 90%) and distinct nutritional health benefits attributed to their high contents of dietary fiber, vitamins (folates, A, B, and C), and essential minerals (K, Ca, Fe, Mg, Mn, P, and Zn), unlike dry beans whose nutritional qualities are predominantly characterized by proteins, carbohydrates, and soluble fibers (Janssen et al. 1988; Myers et al. 2019; Chaurasia 2020). In addition, snap bean pods contain phenols and flavonoids, which are two families of molecules that are well-known for their antioxidant activities (Abu-Reidah et al. 2013).

Pod phenotypes are diverse and key in the grouping of snap beans, where various market classes or phenotypic groups are established based on phenotypic variations (García‑Fernández et al. 2022). Pod phenotypic diversity primarily includes variations in color (pod color and patterns) and characteristics associated with pod morphology (length, width, cross-sectional shape, curvature, and pod beak shape). Genetic control of pod morphological phenotypes has been the focus of classical genetic studies on common bean, and consequently, certain Mendelian genes regulating pod morphological traits (PMTs) have been identified. For example, changes in pod cross-sectional shape are attributed to four different genes, where the dominant alleles (*Ea* and *Eb*; *Ia* and *Ib*) result in elliptical pod phenotypes and recessive alleles (*ea* and *eb*; *ia* and *ib*) regulate round pod phenotypes (Tschermak 1916; Lamprecht 1932a, b, 1947, 1961). Regarding pod length, the recessive allele of the *Ds* gene is associated with short pods with deep constrictions between the seeds (Bassett 1982). Two genes, *Da* and *Db*, which were described by Lamprecht (1932b, 1947), are associated with straight pod regulation. Nevertheless, recent studies have investigated the genetic regulation of PMTs using quantitative inheritance models. Studies conducted on biparental populations and genome-wide association studies (GWAS) have reported major and minor quantitative trait loci (QTL) for PMTs across 11 common bean chromosomes (see review Nadeem et al. 2021). However, to date, the major genes associated with the genetic regulation of PMTs have not been mapped and most of the associated QTL have not been validated across different genotypes and environments, which are crucial features for precision breeding.

In addition to pod morphology, other pod characteristics, such as insoluble fiber content, the temporal window of development in which characteristics of pods is preserved, seed size, seed development rate, and flavor, also affect snap bean pod quality and determine their use as fresh vegetables or frozen/processed foods (Silbernagel 1986; Leakey 1988; Cortinovis et al. 2021). Snap beans are presumably derived from dry beans through a stepwise process of domestication and breeding: (i) reduction of pod wall fiber, (ii) more succulent pods, (iii) varying morphological shapes, (iv) different pod colors, and (v) absence of suture strings (Myers and Baggett 1999; Wallace et al. 2018). The reduction of pod wall fiber is a major step because it is the only step that conditions pod edibility and therefore, constitutes a distinguishing characteristic between dry and snap beans. The remaining evolutionary steps are regarded as improvement traits aimed at increasing snap pod quality. A typical example is the absence of suture strings in pods of modern snap bean varieties. Conventional snap bean varieties have pod suture strings that should be removed before cooking or processing to avoid reducing the sensory quality of the pods. Thus, the identification and development of stringless varieties meant an improve in the quality of snap bean pods by facilitating their processing.

With regard to the regulation of specific pod traits associated with snap beans, various studies have proposed different hypotheses based on the inheritance model that underlies the regulation of pod wall fiber deposition. The simplest model suggests the involvement of a single gene model with wall fiber dominant over no wall fiber (Emerson 1904; Tjebbes and Kooiman 1919, 1922; Wellensiek 1922; Prakken 1934; Atkin 1972). Furthermore, Wade and Zaumeyer (1940) hypothesized a two-gene model but in this case, the control would be exercised by two complementary genes. The most complex classical model proposed is based on a three-gene model composed of a basic gene (*Fa*) and two supplementary genes (*Fb*, *Fc*) that would act as modifiers (Lamprecht 1932b, 1947). Koinange et al. (1996) were the first to map a gene associated with the absence of pod wall fibers on chromosome Pv02 using a biparental population derived from a cross between the cultivar “Midas” and wild accession G12873. Subsequently, Hagerty et al. (2016) described a new QTL for pod wall fiber located on chromosome Pv04 using another dry bean x snap bean recombinant inbred population (OSU5446 x RR6950). Nevertheless, the low level of fiber deposition in the pod wall and pod sutures in snap beans have been shown to be correlated with extreme resistance to pod dehiscence (Parker et al. 2021a). In this regard, certain QTL for bean pod indehiscence have been identified on Pv02, Pv03, Pv04, Pv05, Pv08, and Pv09. Various candidate genes within these genomic regions have been proposed, including *PvIND* (Pv02), the common bean ortholog of INDEHISCENT, *PvPdh1* (Pv03), which is described as a major locus controlling pod shattering in common bean, NAC family transcription factors (Pv03), C2H2-type zinc finger (Pv03), MYB family transcription factors, such as *PvMYB26* and *PvMYB46* (Pv05 and Pv08), WRKY family transcription factors (Pv08), polygalacturonases (Pv08 and Pv09), and cellulose synthase (*CESA7*) (Pv09) (Rau et al. 2019; Parker et al. 2020; 2021b; Di Vittori et al. 2021; Gioia et al. 2013).

In summary, pod phenotype is a complex characteristic defined by a combination of various traits whose genetic control depends on a complex network of major genes and QTL, which interact to build the final pod phenotype. The current study aimed to identify the genomic regions involved in the genetic control of relevant PMTs and edible pod characteristics (EPC) in a recombinant inbred line (RIL) population with extreme pod phenotypes, which was derived from a cross between the cultivars ‘TU’ (dry bean) x ‘Musica’ (snap bean). The results of this study contributed to the consolidation of existing knowledge on the genetic control of pod morphology and potential consumption as a snap bean, a key tool for the implementation of future targeted breeding programs.

## Material and methods

### Plant material

The mapping population used in this study (TUM population) was established using 175 recombinant inbred lines (RILs, F_6:7_) obtained by single-seed descent from a cross between the cultivars ‘TU’ (female parent) and ‘Musica’ (male parent). These parental lines were selected based on extreme pod phenotypes (Figure S1). The parent ‘TU’ is a well-known dry cultivar for its resistance to anthracnose disease and its pod phenotype is characterized by short and narrow pods, while the parent ‘Musica’ is a snap bean cultivar type ‘Romano’ with extra-long, wide, and flat pods (Figure S1). Both parents have indeterminate growth habits and are related to the Mesoamerican gene pool, but with different Andean introgression levels (Campa et al. 2018). The RIL population has a genetic linkage map composed of 842 highly informative single nucleotide polymorphism (SNP) markers obtained by genotyping-by-sequencing (GBS) (García‑Fernández et al. 2021b; data available at 10.5281/zenodo.5962114). SNP markers were named according to their physical position in the bean reference (G19833) genome sequence (v2.1) (https://phytozome-next.jgi.doe.gov) taking into account the chromosome and physical position in base pairs (e.g., S07_28535059).

A subset of the Spanish Diversity Panel, SDP (Campa et al. 2018) composed of 137 lines that was well-characterized as snap bean (*N* = 69) and dry bean (*N* = 68) from passport data was used to verify the involvement of putative regions identified in this study. Old and elite snap bean cultivars, as well as the parental lines ‘TU’ and ‘Musica’ were included in the selected lines (Table S1). The set of lines was genotyped with 8267 SNP markers obtained by GBS after filtering for missing values (< 5%) and minor allele frequency (MAF > 0.05) (data available at 10.5281/zenodo.7003990).

### Experimental design

The TUM RIL (F_6:7_) population and parental lines were grown and evaluated in greenhouses at the Regional Agrifood Research and Development Service (SERIDA), Villaviciosa, Asturias, Spain (43° 29′01’N, 5° 26′11’W; elevation 6.5 m). For PMTs, the RIL population and parental lines were evaluated in four consecutive trials (autumn 2018, spring 2019, autumn 2019, and spring 2020), whereas EPC was tested in three trials (autumn 2019, spring 2020, and spring 2021). The spring season included crops from March to July, while the autumn season included crops from August to November. Each plot had a single 1 m row with 8–10 plants per recombinant inbred line. A randomized design with one plot per line was used for all greenhouse trials. The seeds were germinated in trays containing peat and then transplanted to ensure homogeneity of the crop. Standard agronomic practices for tillage, fertilization, and weed and insect control were followed to ensure plant growth and development. Finally, 10 F_1_ plants were grown in a single trial during the summer of 2023.

### Pod phenotyping

Phenotypic characterization of pods in the TUM RIL population was carried out based on six traits associated with pod morphology: pod dimensions [maximum pod length (PL), maximum pod width (PLW), and thickness (PTH)], shape or fit of the cross-section to circularity [pod cross-sectional height (PSH)/pod cross-sectional width (PSW) index], number of seeds per pod (NSP), and seed weight (SW). Ten pods per line were harvested at the beginning of the R8 stage (mid-pod filling stage or seed growth) and their morphometric traits were measured using Tomato Analyzer v3 (Rodríguez et al. 2010) with an image resolution of 200 and 750 dpi for pod dimensions and cross-section measurements, respectively (Figure S1). At the end of the cycle (dry pod stage), NSP was determined by counting the average number of seeds per pod on a random subsample of 10 pods and SW was determined by weighing four replications of 25 seeds each.

EPC was qualitatively evaluated as the edibility of immature pods at the commercial stage (beginning of R8 stage). Six to ten pods per line were harvested, cut into pieces (3 cm long), and weighed. A random sample of 50 g per line was cooked for 25–30 min at 95 °C in fine labeled cotton bags. The parent ‘Musica’ in all batches was used as an optimal cooking time control. Parental lines were used as positive (‘Musica’) and negative (‘TU’) control for the pod characteristics. The sensory quality of pods was evaluated by a three-member panel trained for cooked snap bean quality analysis. In each season, 10–15% of the RIL lines that were randomly sampled were evaluated twice to test the repeatability of the evaluation. Finally, each RIL line was qualitatively classified as ‘edible’ (quality snap bean with succulent tender pods and reduced insoluble fibers like parent ‘Musica’) or ‘nonedible’ snap bean (non-quality snap bean with hard and fibrous pod walls like parent ‘TU’).

### Statistical analyses

All statistical analyses were performed using R v4.0.3 (R Core Team 2020) based on a significance threshold of *α* ≤ 0.05. First, data outliers were removed based on the interquartile range (Tukey 1977). Descriptive statistical analyses (chi-square, Fisher’s, and *t*-tests) of phenotypic data and statistical analyses were computed using the “Rcmdr” package in R (Fox 2005). The overall mean adjusted value of each quantitative trait for each RIL line was computed using the least squares method with the lsmeans package in R (Lenth 2016). The frequency distribution of each quantitative morphological trait was visualized using the hist() function in R. The goodness-of-fit for normal distribution was tested using Kolmogorov–Smirnov test and homogeneity of variances was determined using Levene’s test. The Yeo-Johnson transformation was performed in the case of traits that did not conform to normal distribution using the “Johnson” package in R (Santos 2014). Statistical comparisons between groups were determined using Student’s *t*-tests and the Mann–Whitney-Wilcoxon’s test depending on whether the data were or not normally distributed. The genetic correlation networks between traits were determined using Pearson’s correlation coefficient of the adjusted and normalized means with the “corrplot” package in R (Wei and Simko 2017). The broad-sense heritability (*H*^*2*^) for each quantitative trait was estimated using the repeatability function of the ‘heritability’ package (Kruijer et al. 2015). *H*^*2*^ was estimated at the genotypic level according to the following equation: *Vg/(Vg* + *Ve/r)*, where *Vg* = *[MS(G)–MS(E)]/r, Ve* = *MS(E)*, where *r* represents the number of replicates per genotype, *MS(G)* represents the mean sum of squares for genotype, and *MS(E)* represents the mean sum of squares for residual error obtained from the analysis of variance.

### QTL mapping

The available genetic linkage map (García‑Fernández et al. 2021b) and the adjusted and normalized phenotypic data of each morphological trait were used to detect QTL. QTL analysis was conducted using the composite interval mapping (CIM) method implemented in QGene v.4.4.0 (Joehanes and Nelson 2008). QTL scan interval was set to 2 cM. Significant thresholds for QTL detection were fixed through the generation of 1000 permutation tests at *α* = 0.01. Additive effect and percentage phenotypic variation attributable to individual QTL (R^2^) were inferred at the point of maximum logarithm of the odds (LOD) score (also called QTL peak) in the region under consideration. Single QTL with percentages explaining greater than 10% of the phenotypic variance were considered. The nomenclature of the QTL was based on the abbreviation of the trait, linkage group number, serial number, and the abbreviation of the genetic background (in superscript) from which it has been inferred (e.g., PL1.1^TUM^) according to Miklas and Porch (2010) guidelines. The position of detected QTL on the genetic linkage map was graphically represented using MapChart v.2.32 (Voorrips 2002).

### Colocation with previously described QTL

The QTL identified in the TUM mapping population was compared to previously reported QTL regions in the common bean for each of the morphological traits evaluated in this study. Detailed information regarding the studies retrieved for this comparative analysis is summarized in Table S2. The physical positions of the reported QTL regions were established by aligning their flanking or underlying markers with the bean reference (G19833) genome sequence v2.1 by performing a BLASTN search (https://phytozome-next.jgi.doe.gov/blast-search). Marker sequences were obtained from the literature, the Legume Information System (https://legumeinfo.org) (Dash et al. 2016), and the Pulse Crop Database Resources (https://www.pulsedb.org). Genomic regions with consensus physical positions were established and labeled as consensus QTL for common bean PMTs based on the overlapping positions between QTL mapped from independent studies. The nomenclature of the consensus QTL was based on ‘Pod’ (referring to pod traits) followed by the linkage group number, serial number, and the abbreviation of *Phaseolus vulgaris* in superscript (e.g., Pod1.1^Pv^).

### Genetic analysis of EPC

The inheritance of EPC in the TUM population was investigated using the goodness-of-fit of observed to expected ratios tested using chi-square tests (*χ*^2^) at *α* ≤ 0.05. To map the genes involved in the control of qualitative EPC, contingency chi-square tests were conducted for the joint segregation of the characteristics and SNP markers included in the genetic map. A significant deviation from random segregation suggested that the chromosomal region tagged with the SNPs was involved in the genetic control of the characteristic. Bonferroni correction was used for multiple comparison corrections at *α* = 0.05 (Bonferroni 1936).

To verify the involvement of the regions identified in the genetic analysis of EPC, a single-locus-GWAS based on a mixed linear model (MLM) was conducted in Tassel v5.1 (Bradbury et al. 2007) on a subset of well-characterized SDP lines as ‘snap’ and ‘non-snap’ beans. Principal component analysis (*N* = 3) and kinship matrix, obtained by the centered-IBS method, were estimated using Tassel v5.1 (Bradbury et al. 2007) to account for multiple levels of relatedness within the lines included in the panel. A critical threshold of significance was set after adjusting the false discovery rate (FDR) for multiple testing corrections using the R package qvalue (Storey et al. 2022). Manhattan and quantile–quantile (QQ) plots were generated using the qqman package in R (Turner 2018). To verify the robustness of the trait-SNP association (quantitative trait nucleotide, QTN), chi-square, and Fisher’s tests were conducted to determine significant differences between groups (‘snap’ and ‘non-snap’) and SNP genotype. Finally, linkage disequilibrium (LD) between SNP markers was analyzed by Haploview v4.2 (Barrett et al. 2005) using the standardized disequilibrium coefficient (D’) to establish haplotype blocks. LD-based haplotype blocks were defined by the confidence interval methods (Gabriel et al. 2002) implemented in Haploview.

### Gene ontology enrichment analysis for PMTs and EPC

Annotated genes underlying each consensus QTL for PMTs and the genomic regions associated with EPC detected in the SDP were explored using the PhytoMine tool in Phytozome v13 (https://phytozome.jgi.doe.gov/phytomine/begin.do). Gene Ontology (GO) biological process enrichment analysis for PMTs and EPC was performed using the clusterProfiler package in R (Yu et al. 2012; Wu et al. 2021).

## Results

### Phenotypic variation, correlation, and heritability of PMTs

**Fig. 1:**
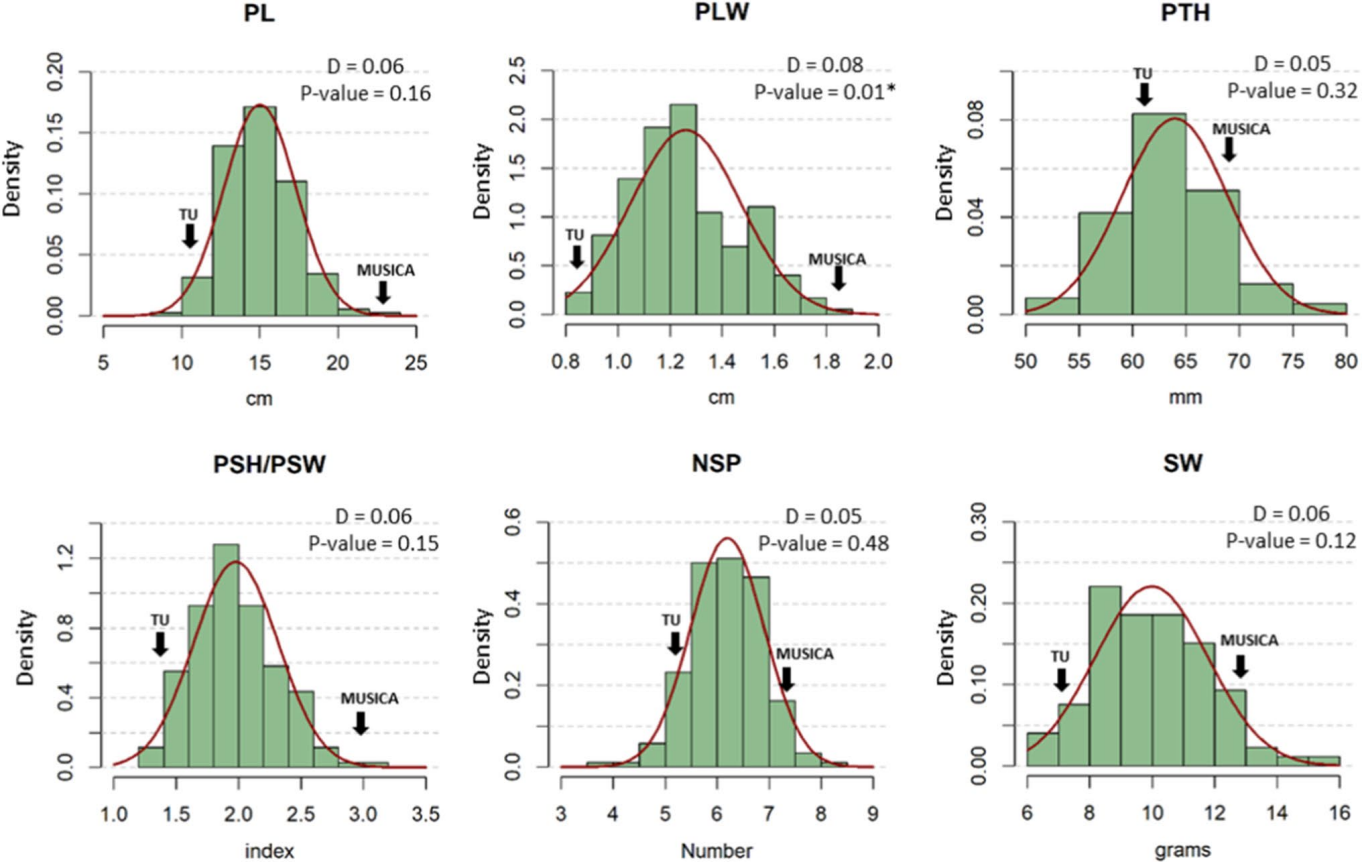
Phenotypic frequency distribution of adjusted means of the six PMTs evaluated in the TUM population. Black arrows indicate the mean phenotype values of the corresponding parent lines. The solid red line represents the normal distribution curve. The results of the normality test (Kolmogorov–Smirnov) are displayed to the right of each histogram

According to the results, 172 TUM RILs were phenotyped for six PMTs (PL, PLW, PSH/PSW, PTH, NSP, and SW). TUM RIL population showed a wide phenotypic variation of all traits evaluated (Figure S2). All traits in the TUM RIL population, except PLW, exhibited a continuous and normal distribution (Fig. 1). The mean phenotypic values for the PMTs evaluated in the parental lines, F_1_ plants, and the corresponding mean and range values in the TUM population are presented in Table 1. Significant differences were observed between parental lines. F_1_ plants exhibited an intermediate phenotype for PMTs that differed significantly from both parental lines for all traits, except for PTH and NSP, which did not exhibit significant differences when compared to the parental line ‘Musica’(Table 1). A comparison between parents and RILs based on the maximum and minimum values revealed the existence of significant transgressive segregations for PTH, NSP, PSH/PSW, and SW (Fig. 1; Table 1). Transgressive segregations were not observed for PL and PLW in which the parental line ‘Musica’ had the highest values. The estimated *H*^*2*^ values of all traits were high, with values ranging from 0.88 (NSP) to 0.98 (PL, PLW, and PSH/PSW) (Table 1).

**Table 1:** Heritability, mean, and standard deviation values (SD) of PMTs evaluated in the parental lines, F_1_ plants, and the TUM RIL population, and statistical comparison between groups

Trait	H^2^	Parents			F_1_			RILs				
		TU	Musica					Mean ± SD	Max		Min	
		Mean ± SD	Mean ± SD	t^a^	Mean ± SD	t^b^	t^c^		Mean ± SD	t^d^	Mean ± SD	t^e^
PL	0.99	10.29 ± 1.03	24.26 ± 2.07	***	14.58 ± 0.91	***	***	15.06 ± 2.66	22.57 ± 1.43	***	9.78 ± 1.22	ns
PLW	0.99	0.84 ± 0.06	1.93 ± 0.22	***	1.15 ± 0.06	***	***	1.25 ± 0.24	1.83 ± 0.20	*	0.86 ± 0.05	ns^(1)^
PTH	0.95	0.61 ± 0.05	0.69 ± 0.07	***	0.66 ± 0.02	**	ns	0.67 ± 0.08	0.82 ± 0.07	***	0.53 ± 0.05	***
PSH/PSW	0.99	1.49 ± 0.09	2.92 ± 0.29	***	1.22 ± 0.06	***	***	1.96 ± 0.33	2.90 ± 2.90	ns	1.33 ± 0.10	***
NSP	0.88	5.58 ± 0.87	7.34 ± 1.29	***	7.00 ± 0.77	**	ns	6.19 ± 1.43	8.13 ± 1.81	*	3.90 ± 1.16	***
SW	0.97	7.15 ± 0.40	12.97 ± 1.09	***	9.50 ± 0.37	***	***	9.86 ± 2.00	15.06 ± 1.25	***^(1)^	6.15 ± 0.51	***

PL: pod length; PLW: pod width; PSH/PSW: fit of the cross-section to circularity; PTH: pod thickness; NSP: number of seeds per pod; SW: seed weight

^(1)^Mann–Whitney–Wilcoxon test

^a^Comparison between parents

^b^Comparison between ‘TU’ and F_1_

^c^Comparison between ‘Musica’ and F_1_

^d^Comparison between the RIL showing the maximum value and the parent showing the higher value

^e^Comparison between the RIL showing the minimum value and the parent showing the lower value

ns = not significant (*α* > 0.05); *0.01 > *α* < 0.05; **0.01 > *α* < 0.001; ***< 0.001

Phenotypic correlations (r) ranged from 0.16 to 0.86 (Figure S3). The highest significant positive phenotypic correlation coefficients (*r* ≥ 0.75***) were observed among the length (PL), width (PLW), and shape (PSH/PSW) of the pod. SW exhibited weak significant positive correlations (0.25**–0.53***) with all morphological traits evaluated, except for NSP, which exhibited a weak negative correlation (− 0.28***). Furthermore, NSP exhibited a weak positive correlation with PL. PTH was significantly positively correlated with PLW and SW, with r values being < 0.35, but moderately negatively correlated with PSH/PSW (*r* =  − 0.33***).

### Detection of major QTL for PMTs

**Fig. 2:**
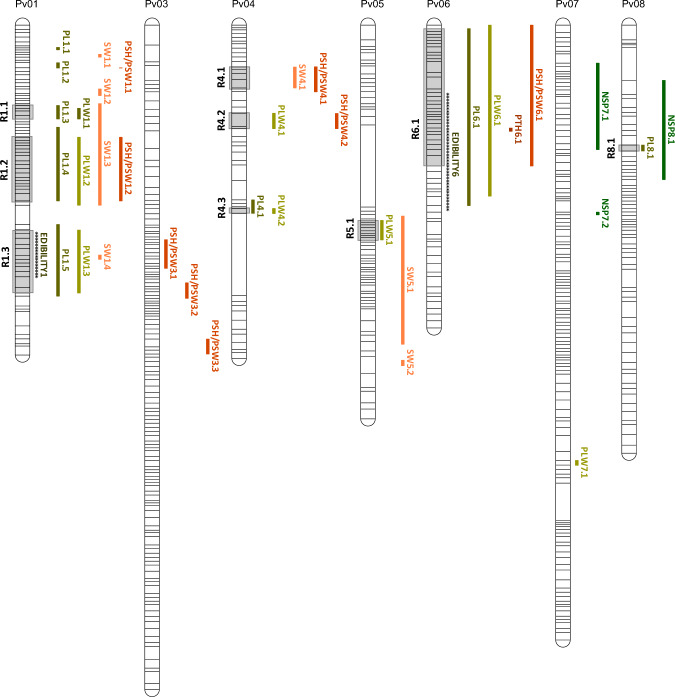
Location of QTL associated with PMTs and genomic regions involved in the control of EPC (EDIBILITY) on the TUM linkage map. QTL are presented as vertical bars on the right of the chromosome. The regions associated with the edible-pod character are indicated as vertical bars of asterisk. The grey boxes indicate the regions with overlapping QTL for PMTs

A total of 35 major QTL were identified for the six pod traits evaluated. The distribution and characteristics of the QTL detected are presented in Fig. 2 and Table 2. Additional information about QTL likelihood curves of LOD scores and box plots diagrams depicting the genetic effect of SNP markers closest to LOD peak score is shown in Figures S4 and S5, respectively. The QTL were distributed on seven linkage groups (Pv01, Pv03, Pv04, Pv05, Pv06, Pv07, and Pv08). The highest number of QTL was located in the linkage group Pv01 with a total of 14 QTL associated with PL, PLW, PSH/PSW, and SW. ‘Musica’ alleles increased pod values in all QTL identified (Table 2). Eight QTL distributed on four chromosomes (Pv01, Pv04, Pv06, and Pv08) were detected for PL, with QTL PL6.1^TUM^ explaining 26% of the phenotypic variation. With regard to PLW, eight QTL located on five chromosomes (Pv01, PV04, Pv05, Pv06, and Pv07) were detected, with QTL PLW6.1^TUM^ explaining 43% of the phenotypic variation. Only one QTL located on Pv06 was detected for PTH. Eight QTL located on four chromosomes (Pv01, PV03, Pv04, and Pv06) were identified for the PSH/PSW ratio and the most significant QTL was that associated with PSH/PSW6.1^TUM^, which explained 22% of the phenotypic variation. Regarding NSP, three QTL explaining 11–14% of the phenotypic variation and located on Pv07 and Pv08 were identified. Seven QTL associated with SW and explaining 10–16% of the phenotypic variation were located on Pv01, Pv04, and Pv05.

**Table 2:** QTL for PMTs detected in the TUM RIL population using the composite interval mapping (CIM) method implemented in QGene v.4.4.0 (Joehanes and Nelson 2008)

QTL ID	Chr	Physical position		Peak score			
		Start	End	Marker	LOD	Add effect*	*R*^2^
PL1.1^TUM^	Pv01	3,564,650	4,588,916	S01_04588916	4.23	− 0.76	0.11
PL1.2^TUM^	Pv01	5,342,249	5,932,870	S01_05932870	4.40	− 0.80	0.11
PL1.3^TUM^	Pv01	13,959,125	17,164,005	S01_16655932	4.74	− 0.81	0.12
PL1.4^TUM^	Pv01	18,036,040	40,033,192	S01_30773753	5.30	− 0.85	0.13
PL1.5^TUM^	Pv01	44,340,326	48,784,096	S01_47561002	5.76	− 0.89	0.14
PL4.1^TUM^	Pv04	12,499,629	37,813,610	S04_37813610	6.52	− 0.96	0.16
PL6.1^TUM^	Pv06	1,406,198	25,087,749	S06_18115056	11.4	− 1.20	0.26
PL8.1^TUM^	Pv08	5,164,857	5,697,166	S08_05164857	3.93	− 0.74	0.10
PLW1.1^TUM^	Pv01	13,323,695	17,164,005	S01_16655932	4.39	− 0.35	0.11
PLW1.2^TUM^	Pv01	19,939,953	40,291,508	S01_30773753	5.82	− 0.40	0.14
PLW1.3^TUM^	Pv01	44,712,299	48,604,665	S01_46498415	6.31	− 0.41	0.16
PLW4.1^TUM^	Pv04	3,197,615	4,035,868	S04_04035868	5.11	− 0.39	0.13
PLW4.2^TUM^	Pv04	33,121,042	37,813,610	S04_37813610	4.68	− 0.37	0.12
PLW5.1^TUM^	Pv05	7,405,171	20,502,505	S05_07534131	5.35	− 0.39	0.13
PLW6.1^TUM^	Pv06	1,406,198	24,219,605	S06_18115056	21.11	− 0.68	0.43
PLW7.1^TUM^	Pv07	32,975,433	33,463,721	S07_33463721	4.23	− 0.35	0.11
PTH6.1^TUM^	Pv06	18,115,056	18,368,785	S06_18115056	4.13	− 0.02	0.11
PSH/PSW1.1^TUM^	Pv01	5,932,870	6,127,306	S01_05932870	4.14	− 0.10	0.11
PSH/PSW1.2^TUM^	Pv01	19,939,953	40,033,192	S01_26300416	5.55	− 0.11	0.14
PSH/PSW3.1^TUM^	Pv03	6,337,778	11,408,730	S03_06337778	4.43	− 0.10	0.11
PSH/PSW3.2^TUM^	Pv03	12,460,987	28,684,062	S03_28684062	4.77	− 0.10	0.12
PSH/PSW3.3^TUM^	Pv03	29,142,137	30,776,734	S03_29142137	4.33	− 0.10	0.11
PSH/PSW4.1^TUM^	Pv04	1,593,169	2,409,491	S04_02205922	5.02	− 0.10	0.13
PSH/PSW4.2^TUM^	Pv04	3,197,615	4,035,868	S04_04035868	5.62	− 0.12	0.14
PSH/PSW6.1^TUM^	Pv06	1,406,198	22,105,330	S06_18115056	9.40	− 0.14	0.22
NSP7.1^TUM^	Pv07	762,958	3,044,819	S07_02095678	5.71	− 0.28	0.14
NSP7.2^TUM^	Pv07	6,390,689	6,922,504	S07_06922504	4.37	− 0.24	0.11
NSP8.1^TUM^	Pv08	1,505,041	8,337,695	S08_03183715	5.06	− 0.26	0.13
SW1.1^TUM^	Pv01	3,723,813	4,006,212	S01_04006212	4.73	− 0.62	0.12
SW1.2^TUM^	Pv01	9,748,863	10,141,735	S01_10141735	4.08	− 0.59	0.10
SW1.3^TUM^	Pv01	12,983,527	40,291,508	S01_30773753	6.01	− 0.71	0.15
SW1.4^TUM^	Pv01	46,498,415	47,167,906	S01_46498415	4.13	− 0.59	0.11
SW4.1^TUM^	Pv04	1,593,169	2,409,491	S04_02205922	5.54	− 0.68	0.14
SW5.1^TUM^	Pv05	7,195,409	36,537,155	S05_24888566	6.44	− 0.74	0.16
SW5.2^TUM^	Pv05	37,885,482	37,923,737	S05_37885482	4.45	− 0.63	0.11

*PL* pod length, *PLW* pod width, *PSH/PSW* fit of the cross-section to circularity, *PTH* pod thickness, *NSP* number of seeds per pod, *SW* seed weight

*Negative values are provided by the parental ‘Musica’

Generally, QTL associated with correlated traits showed colocation on the genetic map, except for NSP (Pv07 and Pv08), which showed a more independent distribution of the rest of the traits (Fig. 2). Such QTL colocation was particularly high on chromosomes Pv01 [regions labeled as R1.1 (13.95–17.16 Mpb), R1.2 (19.93–40.03 Mpb), and R1.3 (44.71–48.60 Mbp)] and Pv06 [region labeled as R6.1 (1.40–24.21 Mbp)] (Fig. 2). QTL associated with PL, PLW, and SW traits in regions R1.1 and R1.3 overlapped, whereas QTL in region R1.2 and the three traits PSH/PSW were colocated (PL1.4^TUM^, PLW1.2^TUM^, SW1.3^TUM^, and PSH/PSW1.2^TUM^). In the case of region R6.1, QTL associated with PL, PLW, PSH/PSW, and SW overlapped (PL6.1^TUM^, PLW6.1^TUM^, PTH6.1^TUM^, and PSH/PSW6.1^TUM^). Moreover, QTL in region R6.1 had the same peak score (peak at 18,115,058 bp) and they accounted for the highest percentage of phenotypic variation of each trait, which ranged from 11% (PTH6.1^TUM^) to 43% (PLW6.1^TUM^) (Table 2).

### Colocalization with previously reported QTL

A total of 289 QTL associated with the genetic control of PMTs were obtained from 23 studies to explore their co-localization with the QTL identified in the TUM population (Table S2). The previously reported QTL were mapped in 11 bean chromosomes, although the physical position of some of the QTL on the genome could not be inferred due to the type of marker used (i.e., RAPD, AFLP, ISSR), the absence of information regarding the markers, or due to mismatches of the physical positions between different versions of the reference genome. The alignment between the reported QTL and the QTL detected in the TUM population revealed 12 overlapping genomic regions located on chromosomes Pv01, Pv04, Pv05, Pv06, and Pv07, which included QTL associated with all the evaluated traits, except for PTH (Table S3). QTL in regions R1.2, R1.3, and R6.1 were repeatedly associated with PMTs based on analyses using biparental populations and diversity panels (Table S3).

Based on the alignment of QTL identified from independent studies and different genetic backgrounds, the common regions between QTL (consensus regions) were inferred, and a total of 15 consensus genomic regions for PMTs in common bean were established. Detailed information regarding the consensus QTL identified are provided in Table 3. Eight of the consensus regions were located on chromosomes Pv01, two on Pv04 and Pv06, and a single region on chromosomes Pv05, Pv07, and Pv08. The sizes of these regions ranged from 38,255 bp (Pod5.1^Pv^) to 5,566,707 bp (Pod1.1^Pv^). Among the consensus QTL located in the genomic regions associated with various PMTs, only Pod4.1^Pv^ was exclusively associated with PL and Pv5.1^Pv^ was associated with SW. Eight of the consensus QTL identified in this study were located in regions R1.2 (Pod1.1^Pv^, Pod1.2^Pv^, Pod1.3^Pv^, Pod1.4^Pv^, Pod1.5^Pv^, and Pod1.6^Pv^) and R6.1 (Pod6.1^Pv^ and Pod6.2^Pv^) that were previously tagged in the TUM population for their involvement in the genetic control of multiple PMTs.

**Table 3:** Consensus QTL for common bean PMTs based on the overlapping positions between QTL mapped from TUM population and other independent studies

ID	Chr	Start	End	Num. QTL	Num. studies	Traits^1^	QTLs described	References^2^
Pod1.1^Pv^	Pv01	19,939,953	26,506,660	6	3	PL, PLW, PSH/PSW, SW	R1.2 (This study), PL1.2^GA^, eSW-1^AM^	1, 2
Pod1.2^Pv^	Pv01	26,506,660	27,389,335	7	3	PL, PLW, PSH/PSW, SW	R1.2 (This study), PL1.2^GA^, SW-1^MA^, eSW-1^AM^	1, 2
Pod1.3^Pv^	Pv01	27,389,335	29,932,212	6	2	PL, PLW, PSH/PSW, SW	R1.2 (This study), SW-1^MA^, eSW-1^AM^	2
Pod1.4^Pv^	Pv01	29,932,212	30,514,836	7	3	PL, PLW, PSH/PSW, SW	R1.2 (This study), PL1.1^XB^, SW-1^MA^, eSW-1^AM^	2, 3
Pod1.5^Pv^	Pv01	32,413,315	33,789,966	7	3	PL, PLW, PSH/PSW, SW	R1.2 (This study, PL1.1^XB^, PL1^PP^, PWI1^PP^	3, 4
Pod1.6^Pv^	Pv01	33,789,966	40,033,192	6	2	PL, PLW, PSH/PSW, SW	R1.2 (This study), PL1^PP^, PWI1^PP^	4
Pod1.7^Pv^	Pv01	45,582,871	45,878,761	6	4	PL, PLW, PLA, PLC, PLP, NSP	R1.3 (This study), PL-1^MA^, ePL-1^MA^, PL-1^PP^, PWI1^PP^, PodL01_45.8, NSP-1^MA^	2, 4, 5
Pod1.8^Pv^	Pv01	48,090,873	48,348,176	5	3	PL, PLW, PLA, PLC, PLP	R1.3 (This study), PL-1^PP^, PWI1^PP^, PodLCol01_48	4, 5
Pod4.1^Pv^	Pv04	15,105,934	15,405,934	3	3	PL	PL4.1^TUM^ (This study), PL4.1^GA^,PL4^PP^	1, 4
Pod4.2^Pv^	Pv04	33,121,042	37,813,610	3	2	PL, PLW	R4.3 (This study), PL4^PP^	4
Pod5.1^Pv^	Pv05	37,885,482	37,923,737	3	3	SW	SW5.2^TUM^ (This study), SW-5^MA^, Sw5.3	2, 6
Pod6.1^Pv^	Pv06	18,115,056	18,368,785	5	4	PL, PLW, PTH, PSH/PSW, SW	R6.1 (This study), ePL-6^MA^, PWI6.1^XB^, SW6.1	2, 3, 7
Pod6.2^Pv^	Pv06	18,457,867	18,781,236	6	4	PL, PLW, PSH/PSW, PLA, PLC, PSH, NSP, SW	R6.1 (This study), PWI6^XB^, NSPLS06_18.4, SW6.1	3, 5, 7
Pod7.1^Pv^	Pv07	6,534,445	6,595,218	5	2	PL, PLW, NSP, PLA	NSP7.2^TUM^ (This study), PL7^XB^, PWI7^XC^*, NSP7^XC^, PA7.2^XB^	3
Pod8.1^Pv^	Pv08	5,164,857	5,697,166	3	2	PL, NSP, SW	R8.1 (This study), SW8.3	8

ID: Consensus QTL name. QTL: Number of QTL from which consensus QTL were inferred. Num. studies: number of studies from which consensus QTL were inferred. Traits: PMTs associated with the consensus QTL

^1^*PL* pod length, *PLW* pod width, *PSH/PSW* fit of the cross-section to circularity, *PLA* pod area, *PLP* Pod perimeter, *PLC* Pod curved, *PTH* pod thickness, *PSH* Pod section height, *NSP* number of seeds per pod, *SW* seed weight

^2^[1] Geravandy et al. (2020); [2] González et al. (2016); [3] Murube et al. (2020); [4] Yuste-Lisbona et al. (2014); [5] García-Fernández et al. (2021a), [6] Blair and Izquierdo (2012); [7] Berry et al. (2020); [8] Hoyos-Villegas et al. (2016)

### Mapping of genomic regions associated with EPC

EPC in 163 TUM RILs obtained from at least two greenhouse trials was evaluated after cooking the snap beans. The RILs were classified into three phenotypic classes: ‘edible’ (*N* = 35), like parental ‘Musica’, ‘nonedible’ (*N* = 108), like parental ‘TU’, and inconsistent phenotype (*N* = 20), which included lines indistinctly classified as both edible and nonedible depending on the greenhouse trial. The lines with inconsistent phenotypes were not considered in the statistical analysis. The nonedible phenotype class had hard pods with fibrous walls like parental ‘TU’, whereas the edible phenotype class had tender pods without a fibrous mouth texture like parental ‘Musica’. The observed segregation fitted an expected Mendelian ratio of 1:3 (edible:nonedible, *χ*^2^ = 0.02; *p* = 0.88), suggesting that EPC was determined by two major independent loci with epistatic effect. Chi-square tests of independence with loci that constitute the TUM genetic map revealed significant associations with markers located on chromosomes Pv01 and Pv06 (Figure S6). The region identified on Pv01 (EDIBILITY1^TUM^) was tagged with nine SNPs located between the physical positions 44.87 and 48.08 Mb (Figure S6). The region identified on chromosome Pv06 (EDIBILITY6^TUM^) was tagged with 24 SNPs located between the physical positions 15.1 and 25.74 Mb (Figure S6). According to the results, for the genetic control of EPC in the TUM RIL population, a model based on two independent loci located on Pv01 and Pv06, where ‘Musica’ alleles in both loci are required to express the edible pod phenotype was proposed. Pods produced by F_1_ plants were qualified with the nonedible pod phenotype, indicating that ‘Musica’ alleles associated with the edible pod phenotype in the TUM population have recessive inheritance.

### Relationship between EPC and PMTs

PMTs and EPC exhibited significant associations with PL, PLW, and PSH/PSW ratio (Table S4). TUM lines in the edible phenotype class had greater mean values of PL, PLW, and more elliptical cross-sectional shapes than those in the nonedible phenotype class.

### Validation of genomic regions associated with EPC

**Fig. 3:**
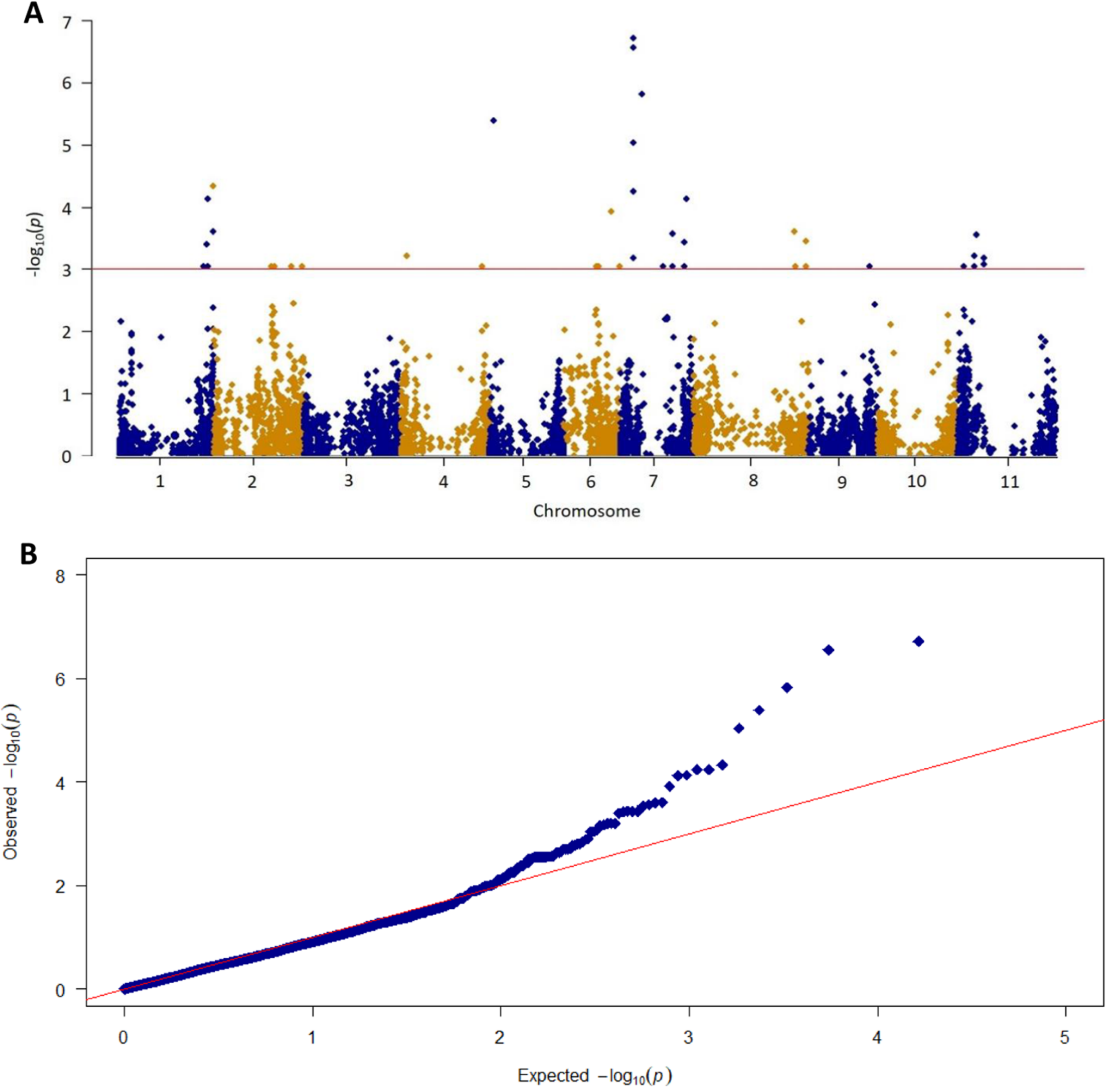
**A** Manhattan and **B** QQ plots of GWAS scan for EPC (‘snap’ vs. ‘non-snap’) on a subset of the SDP using MLM model in Tassel v5.1 (Bradbury et al. 2007). The red line represents the significant threshold (− Log10(*P*) values = 3), which was determined from FDR. Detailed information about the SNP-trait association is shown in the supplementary Table S5

A subset of 137 homozygous lines of the SDP, well-characterized as snap and dry beans, was selected to validate the role of the two genomic regions associated with the genetic control of EPC in the TUM population. GWAS results revealed 59 significant trait-SNP associations (Fig. 3; Table S5), with 40 associations being considered robust (significant Chi-square and Fisher´s exact tests). The significant SNP-trait associations were located on all bean chromosomes, except Pv03 and Pv10. Detailed information regarding robust QTN detected and haplotype blocks established is provided in Table 4. The establishment of haplotype blocks based on LD organized the SNPs into 21 blocks and two single SNP-trait associations (EDIBILITY1.3^SDP^ and EDIBILITY7.2^SDP^). The sizes of the haplotype blocks varied considerably, with the sizes ranging from 23 bp (EDIBILITY2.4^SDP^) to 35 Mb (EDIBILITY11.3^SDP^).

**Table 4:** Robust significant associations detected for EPC (‘snap’ vs. ‘non-snap’) in a subset of the SDP by SL-GWAS with MLM model

QTL ID	SNP ID	Chr	− Log10(*P*)	Block LD			Overlapping with QTL detected in this study
				Number of SNPs	Start	End	
Edibility1.1^SDP^	Chr01pos45729308	Pv01	3	45	45,128,161	46,169,498	Edibility1^TUM^
							PLW1.3^TUM^
							PL1.5^TUM^
							Pod1.7^Pv^
Edibility1.2^SDP^	Chr01pos47721512	Pv01	3	59	47,262,093	48,196,793	Edibility1^TUM^
	Chr01pos48090873		4				SW1.4^TUM^ (near)
							PLW1.3^TUM^
							PL1.5^TUM^
							Pod1.8^Pv^
Edibility1.3^SDP^	Chr01pos50878645	Pv01	4	1	50,878,645	50,878,645	
Edibility2.1^SDP^	Chr02pos21743	Pv02	4	6	21,743	238,928	
Edibility2.2^SDP^	Chr02pos31738490	Pv02	3	67	31,707,543	34,385,471	
	Chr02pos31747845		3				
	Chr02pos31747852		3				
	Chr02pos33626541		3				
Edibility2.3^SDP^	Chr02pos42563539	Pv02	3	60	42,237,266	43,927,166	
Edibility2.4^SDP^	Chr02pos48553125	Pv02	3	3	48,553,125	48,553,148	
	Chr02pos48553136		3				
Edibility4.1^SDP^	Chr04pos43913149	Pv04	3	8	43,901,217	44,049,640	
Edibility5.1^SDP^	Chr05pos2293491	Pv05	5	12	2,215,957	2,357,183	
Edibility6.1^SDP^	Chr06pos18568208	Pv06	3	20	18,368,814	18,934,450	Edibility6^TUM^
							PTH6.1^TUM^ (near)
							PSH/PSW6.1^TUM^
							PLW6.1^TUM^
							PL6.1^TUM^
							Pod6.1^Pv^
Edibility6.2^SDP^	Chr06pos26029176	Pv06	4	9	25,974,122	26,054,074	
Edibility6.3^SDP^	Chr06pos30942196	Pv06	3	36	30,692,356	31,182,173	
Edibility7.1^SDP^	Chr07pos7032967	Pv07	7	40	5,946,397	7,837,485	Pod7.1^Pv^
	Chr07pos7038843		7				
	Chr07pos7149904		5				
Edibility7.2^SDP^	Chr07pos11619102	Pv07	6	1	11,619,102	11,619,102	
Edibility7.3^SDP^	Chr07pos23591893	Pv07	3	7	16,508,071	24,863,366	
Edibility7.4^SDP^	Chr07pos28596434	Pv07	3	37	28,381,719	30,053,416	
Edibility7.5^SDP^	Chr07pos34804331	Pv07	3	57	34,404,090	35,752,849	
	Chr07pos34822110		3				
	Chr07pos34826173		3				
	Chr07pos34826175		3				
	Chr07pos34826212		3				
Edibility7.6^SDP^	Chr07pos36237607	Pv07	4	25	35,752,969	36,328,306	
Edibility8.1^SDP^	Chr08pos55419969	Pv08	4	32	53,557,319	55,427,075	
Edibility9.1^SDP^	Chr09pos34079758	Pv09	3	29	33,745,310	34,228,440	
Edibility11.1^SDP^	Chr11pos3582535	Pv11	3	22	3,294,853	3,659,231	
Edibility11.2^SDP^	Chr11pos9060145	Pv11	3	49	6,390,235	9,082,182	
	Chr11pos9060171		3				
	Chr11pos9060480		3				
	Chr11pos9082087		3				
	Chr11pos9082107		3				
	Chr11pos9082182		3				
Edibility11.3^SDP^	Chr11pos10296440	Pv11	4	91	9,943,844	45,234,144	
	Chr11pos14575556		3				

QTL not overlapping but located at less than 60 Kb were labeled as ‘near’ QTL

*PL* pod length, *PLW* pod width, *PSH/PSW* fit of the cross-section to circularity, *PTH* pod thickness, *NSP* number of seeds per pod, *SW* seed weight, QTL named ‘Pod’ refer to consensus QTL described in this study

Chromosome Pv01 had three haplotype blocks, with two blocks (EDIBILITY1.1^SDP^ and EDIBILITY1.2^SDP^) being located in the EDIBILITY1^TUM^ region. Similarly, three blocks were identified on chromosome Pv06, where EDIBILITY6.1^SDP^ was co-located with the EDIBILITY6^TUM^ region. Therefore, the association analysis validated the role of the two genomic regions previously identified in the TUM population in the genetic control of EPC.

### Potential candidate gene identification for pod phenotype

Genes in the detected regions of PMTs and EPC were explored. Consensus QTL associated with PMT had 730 annotated genes, with 613 genes being functionally annotated (Table S6). GO enrichment analysis revealed a significant overrepresentation of genes (12) associated with the phenylpropanoid metabolic process in the consensus QTL associated with PMTs (Figure S7_A; Table S7). Ten of the genes were located on Pod1.6^Pv^, nine genes were organized in a cluster, and all genes were *DIR* genes encoding dirigent protein 1. Two genes were located on Pod1.5^Pv^ and Pod6.2^Pv^, and both encoded laccase proteins (*lcc13* and *lcc16*). Regarding the EPC (‘snap vs. non-snap’) analyzed in the SDP, the identified regions had 2514 annotated genes, with 2116 genes being functionally annotated (Table S8). In this case, the enrichment analysis revealed a strong overrepresentation of auxin response (53 genes), as well as a slight overrepresentation of the diterpenoid metabolic process (10 genes) (Figure S7_B). Genes with functional annotations associated with auxin response were located in 13 of the genomic regions identified (Table S9). Five of the genes involved in auxin response were located in the genomic regions associated with EPC in the TUM population: EDIBILITY1.1^SDP^ [*Phvul.001G199300* (*aromatic and neutral transporter 1*); *Phvul.001G202000* (*auxin response factor*)], EDIBILITY1.2^SDP^ [*Phvul.001G218700* (*auxin-responsive protein iaa20-related*)], and EDIBILITY6.1^SDP^ [*Phvul.006G071700* (*transmembrane amino acid transporter family protein)*; *Phvul.006G071900* (*transmembrane amino acid transporter family protein)*]. Genes with functional annotation associated with the diterpenoid metabolic process were exclusively located in the EDIBILITY11.3^DP^ region of chromosome Pv11 (Table S10).

## Discussion

Pod phenotype is the result of a combination of several characteristics and a complex gene network, leading to high phenotypic diversity. The traits associated with pod morphology and insoluble fiber content of pod walls in snap beans play a crucial role. Although PMTs determine market classes, consumer preferences, and their uses as fresh or processed food products (Silbernagel et al. 1986; García-Fernandez et al. 2022), the low pod wall fiber deposition enhances the edibility of immature pods, which serves as a distinguishing characteristic between dry and snap beans. In this study, a recombinant inbred population obtained from a cross between dry (‘TU’) and snap (‘Musica’) beans was used to investigate the genetic control of pod phenotypes in the common bean. A mapping population with parental lines that exhibit extreme and contrasting phenotypes is an excellent resource for elucidating the main genetic architecture underlying bean pod morphotypes. In this study, pod phenotype was dissected into PMTS (PL, PLW, PTH, and PSH/PSW, NSP, SW) and EPC.

Phenotyping of PMTs in the TUM RIL population showed a wide, continuous, and normal distribution of all traits evaluated, except for PLW. Significant positive correlations were observed among most of the traits, which is consistent with the findings of previous studies (Hagerty et al. 2016; Murube et al. 2020; García-Fernández et al. 2021a). Similarly, transgressive segregation was observed for traits, including PTH, PSH/PSW, NSP, and SW, which identified recombinant inbred lines that had lower or higher values than those of some parental lines. In contrast to other RIL populations (Hagerty et al. 2016; Geravandi et al. 2020; Pérez-Vega et al. 2010) transgressive segregation was not observed for PL and PLW, indicating that the parental lines ‘Musica’ and ‘TU’ had the most extreme values for both PMTs. All traits showed high heritability (88–98%), which is consistent with the previously reported estimates for common bean (García‑Fernández et al. 2021a; Mesera et al. 2022), indicating that the phenotypic variation observed is controlled by gene expression.

Thirty-five major QTL associated with the six PMTs evaluated were detected and most of them were located on chromosomes Pv01 and Pv06. Considering the overlapping of QTL positions for different PMTs, nine regions distributed across five chromosomes were identified, with three regions (R1.1, R1.2, and R1.3) being located at the end of chromosome Pv01 and one on chromosome Pv06 (R6.1). QTL associated with seed weight, pod dimensions, and shape were located in R1 regions, whereas only QTL associated with pod dimensions and shape were clustered in the R6.1 region. Seed morphology is a key factor influencing pod morphology because the pod is the organ that supports the seeds. Thus, the colocation of genomic regions that control pod and seed morphology is expected because coregulation of both traits ensures the combination of morphotypes. The involvement of chromosome Pv01 in the control of pod morphotypes at the end of the chromosome, where R1 regions are located, could be associated with the role of this region in the regulation of key traits of common bean domestication syndrome, such as determinacy (*Fin/fin* gene) and seed size/weight (Koinange et al. 1996; Kwak et al. 2012; Schmutz et al. 2014, Di Vittori et al. 2017). In addition, the R6 region of chromosome Pv06 was essential because the QTL that accounted for the highest percentage of phenotypic variation (11% for PTH and 43% for PLW) was located in this region. The finding is consistent with the results reported by Davis et al. (2014) for an RI population obtained from a cross between ‘Minutte’ and ‘OSU5630’, where a large region on Pv06 with a major effect on pod phenotype (accounting for 22.9% of the phenotypic variation for PL and 47.6% of the phenotypic variation for PLW) was located.

Nevertheless, the stability and robustness of QTL should be validated using different backgrounds, mapping populations, environments, or breeding programs. The position of QTL identified in the present study was compared to that of previously reported QTL/QTNs. Comparative analysis revealed 12 TUM QTL overlapping with genomic regions already described in the species. The regions R1.2, R1.3, and R6.1 were particularly redundant with those reported previously, which validated their involvement in the control of pod morphology. However, low recombination rates affected biparental populations, in turn, leading to large QTL intervals, as observed in chromosome Pv06 of the TUM population. Nevertheless, the global alignment of QTL previously reported by other authors facilitated the delimitation of common QTL regions inferred from different genetic and environmental backgrounds. In this study, a total of 15 bean consensus QTL for PMTs located on Pv01, Pv04, Pv05, Pv06, Pv07, and Pv08 are described. A recent study mapped 42 meta-QTL associated with phenological parameters and yield components in common bean (Arriagada et al. 2022), where the genomic positions of QTL Yd_MQTL1.2, Yd_MQTL4.2, and Yd_MQTL4.3 overlapped with three consensus QTL (Pod1.8^Pv^, Pod4.1^Pv^, and Pod4.2^Pv^, respectively) and the meta-QTL Yd_MQTL6.2 was located close to the consensus region Pod6.1^Pv^ (approximately 0.3 Mpb). The traits SW and NSP were the primary components of yield (Sinclair 2021); therefore, the association demonstrated the role of these regions in pod morphotypes of the common bean. Notably, the set of genes underlying bean consensus QTL for PMTs revealed an overrepresentation of genes involved in the phenylpropanoid metabolic process. The genes, which were identified by GO enrichment analysis, were located in three of the most crucial genomic regions (R1.1, R1.2, and R6.1) associated with PMTs in the TUM population. Phenylpropanoids are metabolites involved in a wide range of physiological processes, such as flavonoid biosynthesis, lignin biosynthesis, and auxin transport. In this case, the identified genes involved in the phenylpropanoid metabolic process underlying the consensus QTL for PMTs corresponded to *DIR* genes and *lacc* genes, encoding dirigent and laccase proteins, respectively. Both types of genes are strongly involved in the lignin biosynthetic pathway, a natural polymer interlaced with cellulose and hemicellulose in secondary cell walls (Paniagua et al. 2017; Cui et al. 2021), which plays a crucial role in plant growth, tissue/organ development, as well as, response to a variety of stresses (Liu et al. 2018). This result is according to Wang et al. (2023), who in a study on peanut (*Arachis hypogaea* L.) pod size attributed the considerable enrichment of the phenylpropanoid biosynthesis pathway to lignin biosynthesis. Some authors have concluded that the lignification process affects the width of peanut pods, and that ethylene and auxin signaling pathways interact to influence the lignification process and the accumulation of carbohydrates and proteins during the early developmental stages (Wan et al. 2017). Therefore, variations in the phenylpropanoid pathway could affect the phenotype of the plant and organs such as pods (Neutelings 2011).

In the present study, pod wall fiber deposition was evaluated based on EPC in the TUM population. Two major independent recessive loci with epistatic effect were identified for the edible pod phenotype. The genetic model involving two complementary loci is consistent with the inheritance model proposed by Wade and Zaumeyer (1940). Furthermore, the identification of major loci is consistent with a reverse phenomenon referred to as’rogues’ by the industry, where snap bean genotypes lose their edible pod phenotype (return to their ancestral dry pod phenotype) (Parker et al. 2022) because a progressive loss of the phenotype would be expected if the trait was quantitatively inherited. In the case of the TUM mapping population, physical delimitation within the bean genome facilitated location of the first locus between positions 44.8 and 48.0 Mbp on chromosome Pv01 (EDIBILITY1^TUM^), and the second locus between positions 15.9 and 25.7 Mpb on chromosome Pv06 (EDILBILITY6^TUM^). The sizes of the two regions did not allow for the ruling-out of the involvement of more than one locus within each region (linked loci).

The involvement of the EDIBILITY1^TUM^ and EDIBILITY6^TUM^ regions in the control of EPC was validated using a subset of SDP (Campa et al. 2018) composed of snap and dry bean lines. The association analysis between both groups revealed 40 robust labeled SNP-trait associations in 23 genomic regions located on 9 of the 11 chromosomes of the species. The high number of genomic regions inferred from the diversity panel suggested that control of the trait was more complex at the species level. The existence of several regions associated with the control of pod characteristics justified the various models of Mendelian inheritance attributed to the trait based on classical studies (Emerson 1904; Tjebbes and Kooiman 1922; Wellensiek 1922; Prakken 1934; Atkin 1972), as different regions may be involved depending on the genotypic background. Three of the identified regions (EDIBILITY1.1^SDP^, EDIBLILITY1.2^SDP^, and EDIBILITY6.1^SDP^) overlapped with regions in which major loci for EPC (EDIBILITY1^TUM^ and EDIBILITY6^TUM^) were mapped, which highlighted the key role of the positions in the genetic control of the trait. In addition, the overlapping of EDIBILITY1.1^SDP^ and EDIBILITY1.2^SDP^ regions with the EDIBILITY1^TUM^ region is consistent with a previously proposed hypothesis that the regions identified in the TUM population could have more than one locus. Conversely, the other three regions identified by GWAS on the diversity panel (EDIBILITY2.3^SDP^, EDIBILITY4.1^SDP^, and EDIBILITY9.1^SDP^) were close to or overlapping physical positions associated with pod fiber or indehiscent pod QTL previously reported in common bean. Specifically, EDIBILITY 2.3^SDP^ was located close to the genomic region on Pv02 (< 0.1 Mpb), where a pod wall fiber gene called *stringless* (*St*) was described (Koinange et al. 1996; Hagerty et al. 2016), and the common bean ortholog of INDEHISCENT, which is associated with the loss of pod strings (Parker et al. 2022). EDIBILITY4.1^SDP^ was located close to PWF4.1 QTL (< 0.4 Mpb) for pod wall fiber on Pv04, as reported by Hagerty et al. (2016) for which specific candidate genes have not been described. Finally, EDIBILITY9.1^SDP^ overlapped with a major region on Pv09 that is associated with pod indehiscence for which cellulose synthase A7 (*CESA7*) and two polygalacturonases have been proposed as potential candidate genes (Parker et al. 2020).

Furthermore, GWAS results revealed that EDIBILITY1.1^SDP^, EDIBILITY1.2^SDP^, EDIBILITY6.1^SDP^, and EDIBILITY7.1^SDP^ regions colocalized with consensus QTL associated with PMTs (Pod1.7^Pv^, Pod1.8^Pv^, Pod6.1^Pv^, and Pod7.1^Pv^) demonstrated the relationship between pod fiber content and pod morphology. In this regard, edible pods of the TUM population were significantly associated with a pod morphological phenotype characterized by wide and long pods with reduced adjustment to the cross-sectional circularity. These associations are consistent with the results reported by Murgia et al. (2017), who observed that PL and PLW were significantly negatively correlated with pod shattering levels (synthesis of fiber) in common bean, suggesting that the synthesis of biomolecules and tissues required for shattering has an “energy cost”, which may reduce the resources available for seed and pod development, and consequently affect its morphology. In contrast, Hagerty et al. (2016) observed a moderate positive correlation between PLW and pod wall fiber in the RR138 RI population derived from a cross between RR6950 and OSU5446, suggesting that variations in pod wall fiber could directly influence pod shape. Therefore, there seems to be a distinct association between pod morphology (especially PLW) and pod wall fiber deposition in common bean, with certain discrepancies being observed in the association between genotypes.

GO enrichment analysis revealed a strong overrepresentation of genes involved in auxin response in the set of genomic regions for EPC identified from the SPD. Considering the key role of auxins in lignin biosynthesis (Qu et al. 2021), it is highly likely that they are key components associated with pod wall fiber deposition, in turn, influencing the edible pod phenotype. Although PMTs and EPC constitute well-defined pod characteristics, the influence of the lignin biosynthetic pathway on the determination of both characteristics could be attributed to their strong correlation. In addition, the correlation between PMTs and EPC is attributed to the fact that the selective pressure exerted on certain PMTs over time could have led to the selection of varieties with a low content of insoluble fibers in their pods, and consequently, to the development of snap bean varieties.

## Conclusion

The present study revealed the roles of chromosomes Pv01 and Pv06 in the regulation of bean pod phenotypes. The pleiotropic effect of the regions on chromosomes Pv01 and Pv06 on pod wall fiber and pod morphology showed that both traits had a common genetic control, or are controlled by different but linked genes, which were probably associated with the lignin biosynthetic pathway. In addition, 15 bean consensus QTLs for PMTs and 23 bean genomic regions associated with EPC were described. The findings provided novel insights into the genetic control of pod phenotypes in the common bean, which could facilitate the development of future breeding programs targeting pod traits.

### Supplementary Information

Below is the link to the electronic supplementary material.

Supplementary file1 (PDF 1891 kb)

Supplementary file2 (PDF 561 kb)

Supplementary file3 (XLSX 22 kb)

Supplementary file4 (XLSX 69 kb)

Supplementary file5 (XLSX 220 kb)

Supplementary file6 (XLSX 12 kb)

## Data Availability

All data generated or analyzed during this study are included in this published article and its supplementary information files. The genotyping data supporting this study are available at the Zenodo repository: 10.5281/zenodo.5962114.
