# Supplementary material for: Genetic control of pod morphological traits and pod edibility in a common bean RIL population
Source: Theor Appl Genet. 2023 Dec 13;137(1):6. doi: 10.1007/s00122-023-04516-6 (PMC10719158; doi:10.1007/s00122-023-04516-6)

Figure S1. Pod phenotypes of the parental lines of the TUM population. Pod dimensions were measured longitudinally (PL: pod length, and PLW: pod width) and in the cross-section (PSH: pod section height, and PSW: pod section width). A: Scaled longitudinal pod. B: Scaled cross-section pod.

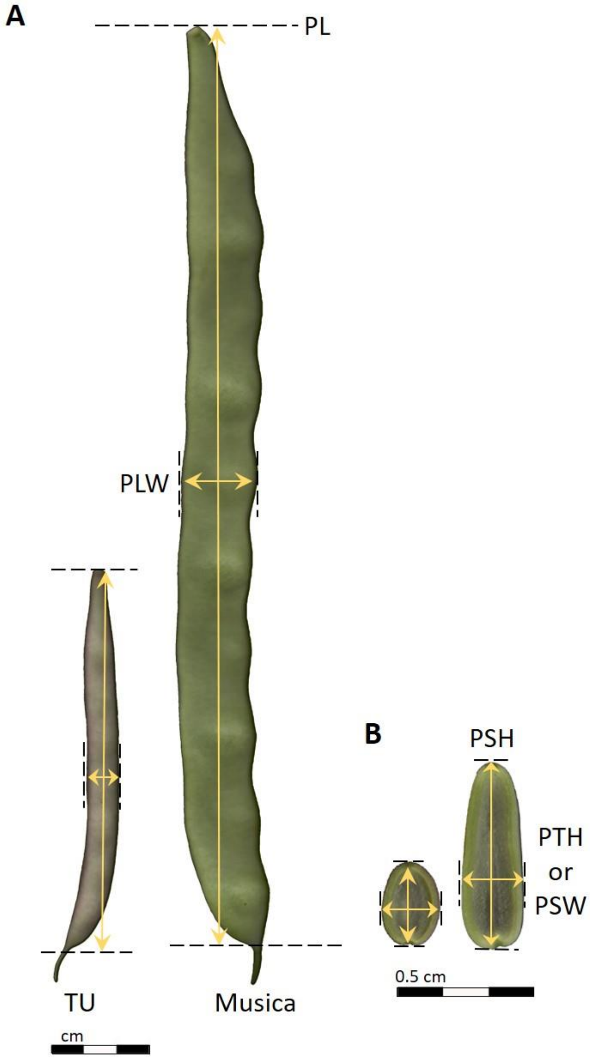

Figure S2. Pods of TUM recombinant inbred line (RIL) population showing phenotypic variation. Each pod is derived from a different recombinant line.

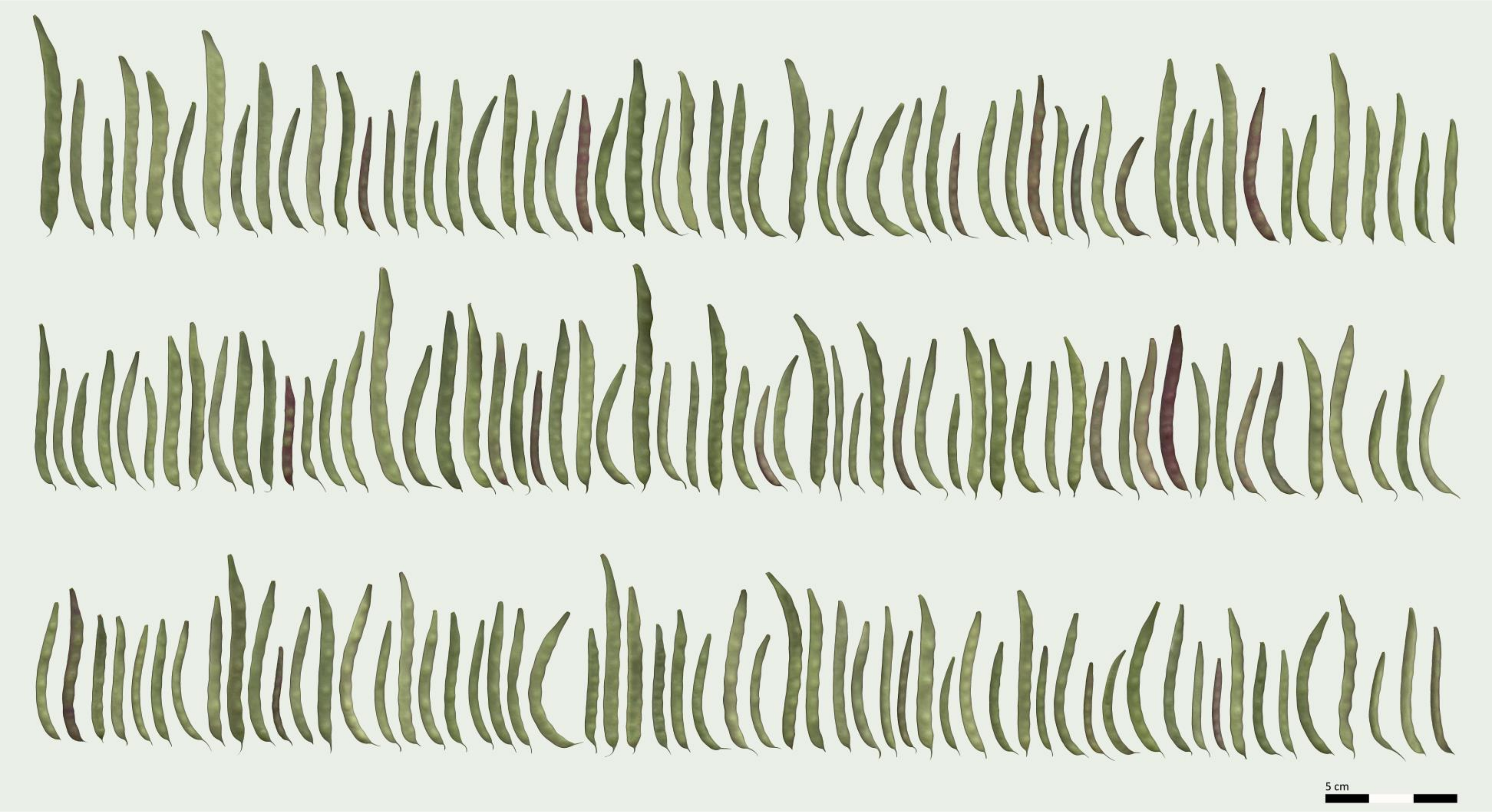

Figure S3. Correlogram depicting the Pearson's correlation coefficients among the six PMTs evaluated in the TUM RIL population (PL: pod length; PLW: pod width; PSH/PSW: fit of the cross-section to circularity; PTH: pod thickness; NSP: number of seed per pod; SW: seed weight). *, **, and *** indicate significant correlations at α = 0.05, 0.01 and 0.01, respectively. Blank cells indicate no significant correlation at α = 0.05.

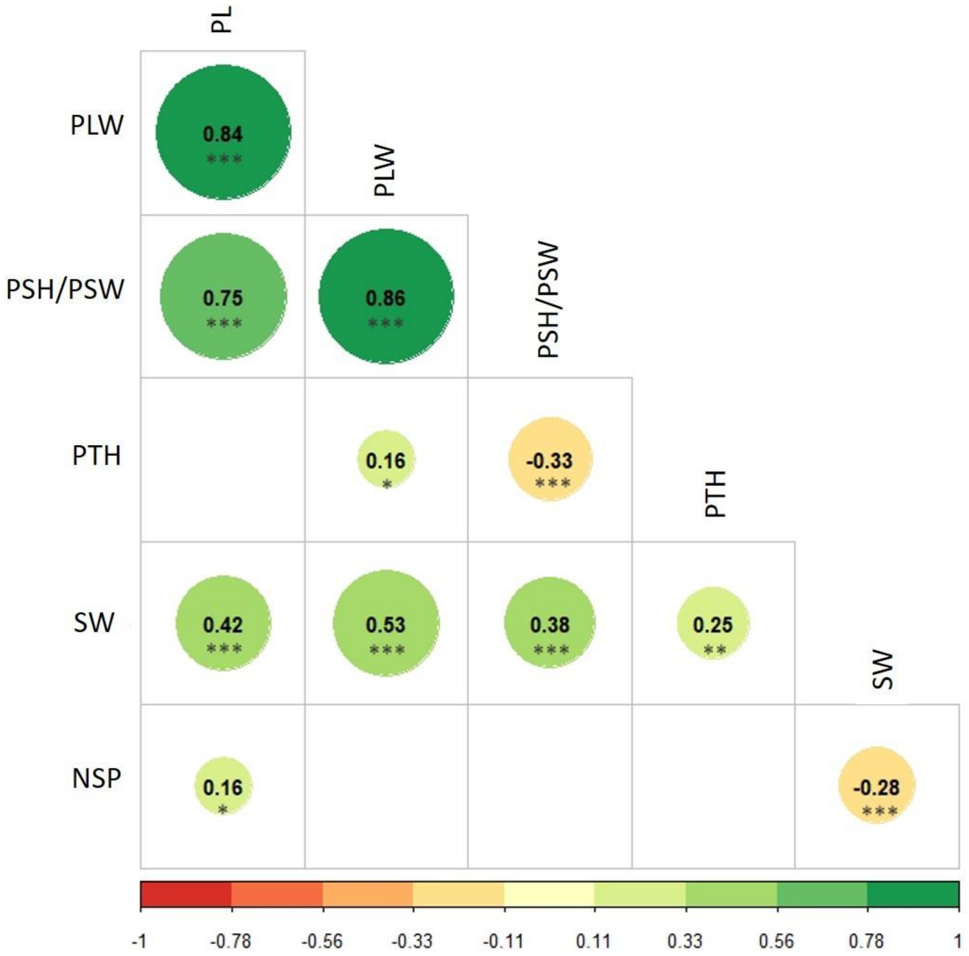

Figure S4. QTL likelihood curves of LOD score for the six PMTs evaluated in the TUM RIL population identified on chromosome Pv01, Pv03, Pv04, Pv05, Pv06, Pv07, and Pv08 through composite interval mapping (CIM) using QGene v.4.40 (Joehanes and Nelson 2008). Horizontal line represents the significant LOD threshold at α = 0.01 after 1,000 iterations in permutation analysis. The traces for individual traits are color coded. PL: pod length; PLW: pod width; PSH/PSW: fit of the cross-section to circularity; PTH: pod thickness; NSP: number of seeds per pod; SW: seed weight.

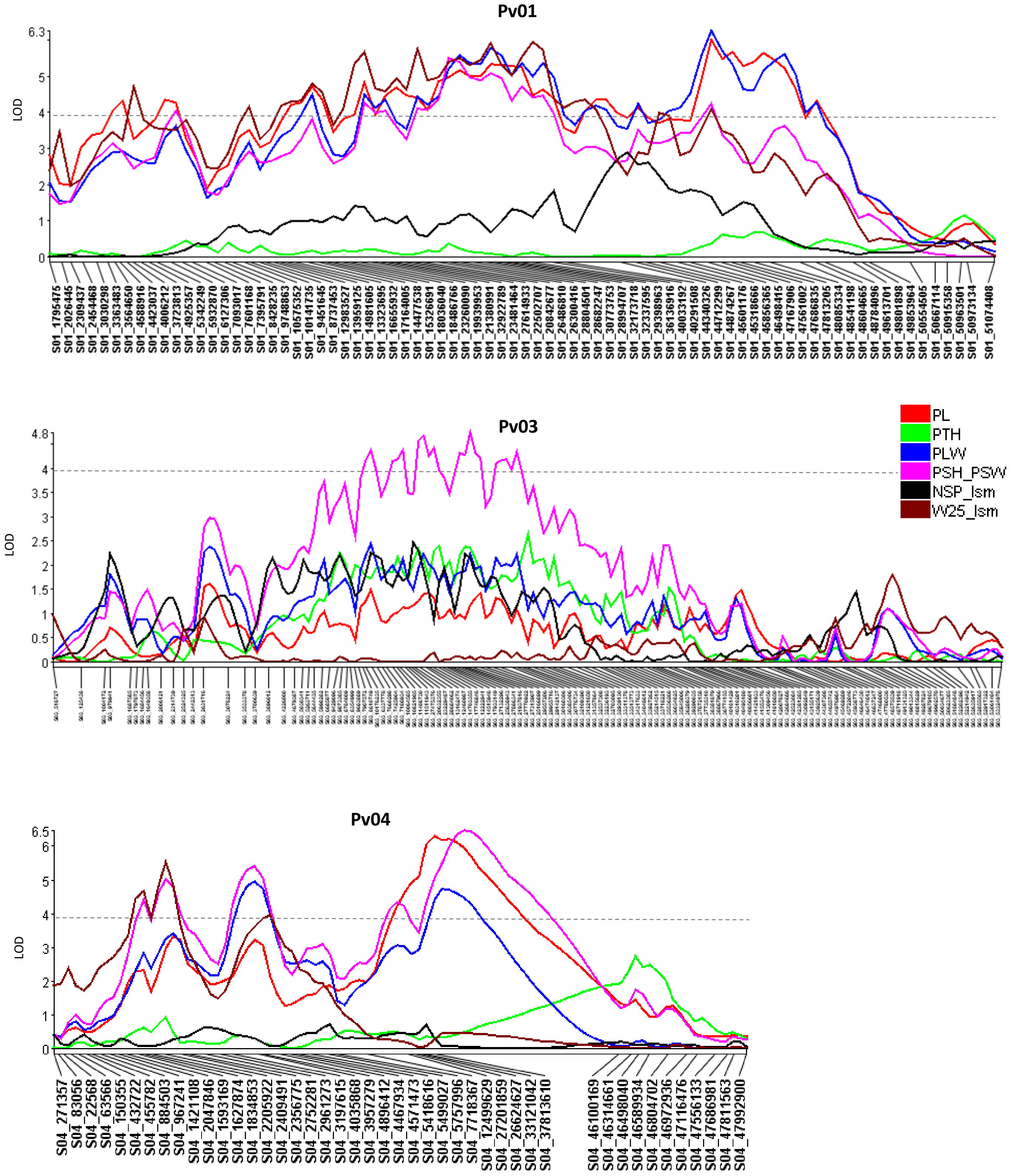

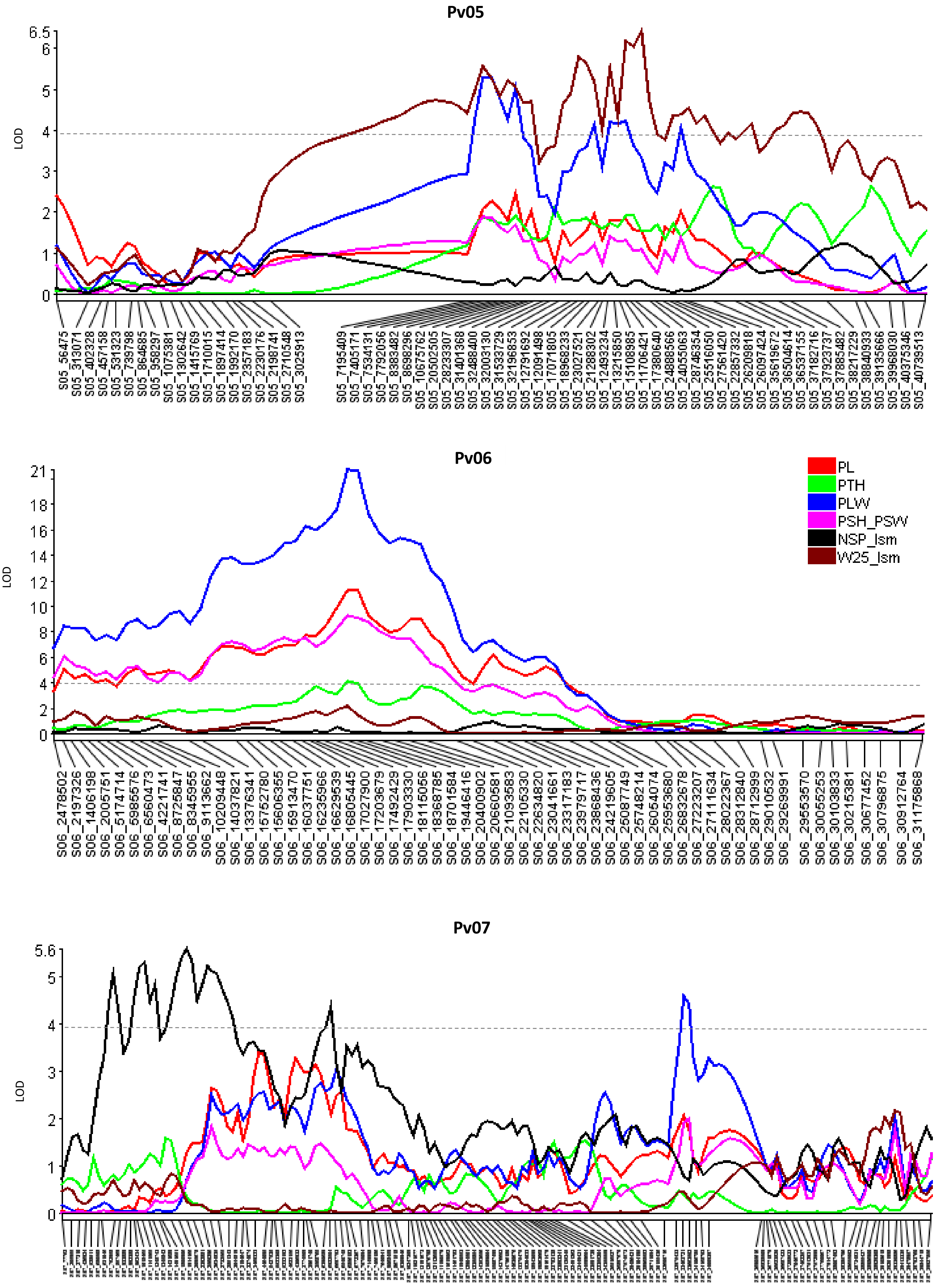
Pv05
LOD
6.5
6
5
4
3
2
1
0
S05_56475 S05_313071 S05_402328 S05_457158 S05_531323 S05_739798 S05_864685 S05_958297 S05_1075381 S05_1302642 S05_1415769 S05_1710015 S05_1897414 S05_1992170 S05_2357183 S05_2230176 S05_2198741 S05_2710548 S05_3025913
S05_7195409 S05_7405171 S05_7534131 S05_7792056 S05_8383482 S05_8698296 S05_10675762 S05_20502505 S05_28233307 S05_31401368 S05_32488400 S05_32003130 S05_31533729 S05_32196853 S05_12791692 S05_12091498 S05_17071805 S05_18968233 S05_23027521 S05_21288302 S05_12493234 S05_13215850 S05_15108985 S05_11706421 S05_17380640 S05_24888566 S05_24055063 S05_28746354 S05_25516050 S05_27561420 S05_22857332 S05_26209818 S05_26097424 S05_35619672 S05_36504614 S05_36537155 S05_37182716 S05_37923737 S05_37885482 S05_38217229 S05_38840933 S05_39135666 S05_39968030 S05_40375346 S05_40739513
Pv06
PL
PTH
PLW
PSH_PSW
NSP_lsm
W25_lsm
LOD
21
18
16
14
12
10
8
6
4
2
0
S06_2478502 S06_2197326 S06_1406198 S06_2005751 S06_5174714 S06_5985576 S06_6560473 S06_4221741 S06_8725847 S06_8345955 S06_9113662 S06_10209448 S06_14037821 S06_13376341 S06_15752780 S06_15606355 S06_15913470 S06_16037751 S06_16235966 S06_16629539 S06_16805445 S06_17027900 S06_17203679 S06_17492429 S06_17903330 S06_18115056 S06_18368785 S06_18701584 S06_19446416 S06_20400902 S06_20660581 S06_21093583 S06_22105330 S06_22634820 S06_23041661 S06_23317183 S06_23979717 S06_23868436 S06_24219605 S06_25087749 S06_25748214 S06_26054074 S06_25953680 S06_26832678 S06_27223207 S06_27111634 S06_28022367 S06_28312840 S06_28712999 S06_29010532 S06_29269991 S06_29553570 S06_30055253 S06_30103833 S06_30215381 S06_30677452 S06_30796875 S06_30912764 S06_31175868
Pv07
LOD
5.6
5
4
3
2
1
0

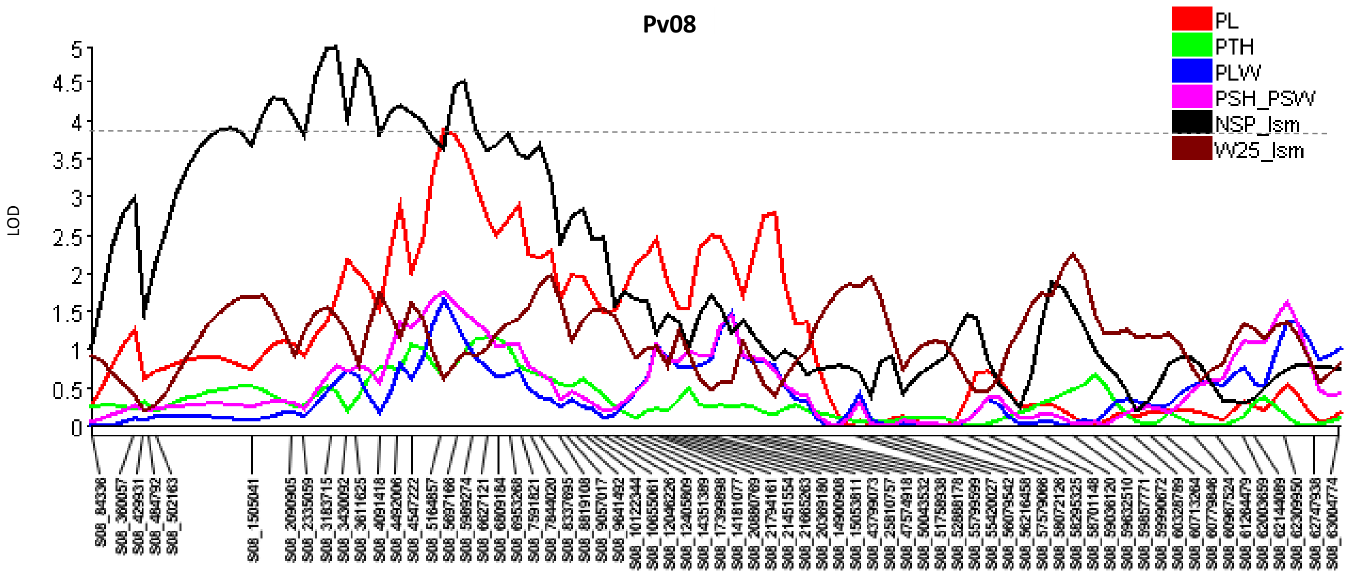

Pv08
PL
PTH
PLW
PSH_PSW
NSP_lsm
W25_lsm
LOD

Figure S5. Box plots diagrams depicting the genetic effect of sentinel SNPs (SNP marker closest to LOD peak score) showing associations with PMTs detected in the TUM RIL population. The X-axis represents the two alleles for each SNP (A: 'TU' allele; B: 'Musica' allele), while the Y-axis corresponds to the PMT phenotype. t: T-student test value; df: degrees of freedom; p-value: level of probability. A: PL (pod length); B: PLW (pod width); C: PSH/PSW (fit of the cross-section to circularity); D: PTH (pod thickness); E: NSP (number of seed per pod); F: SW (seed weight).

A

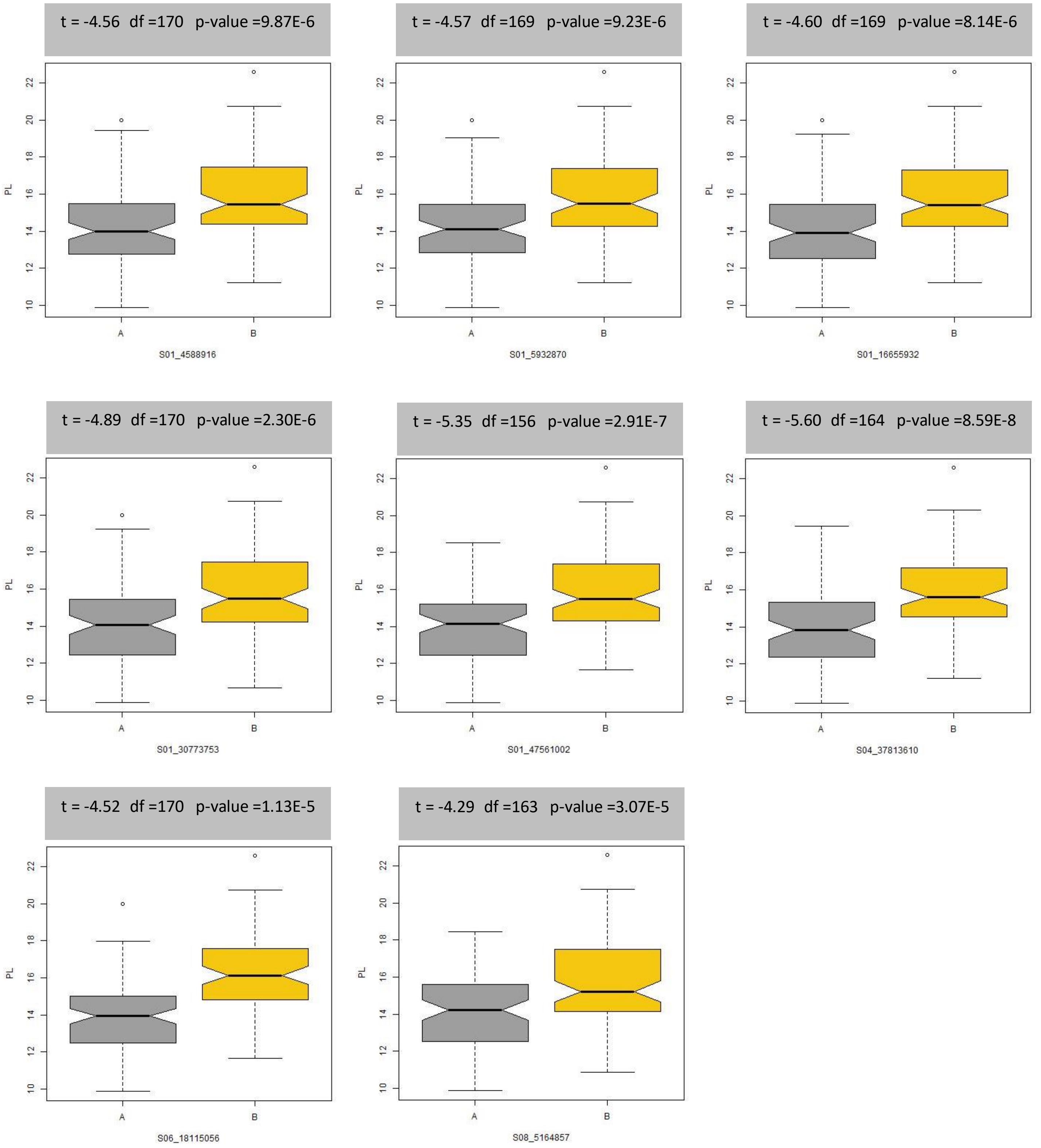

B

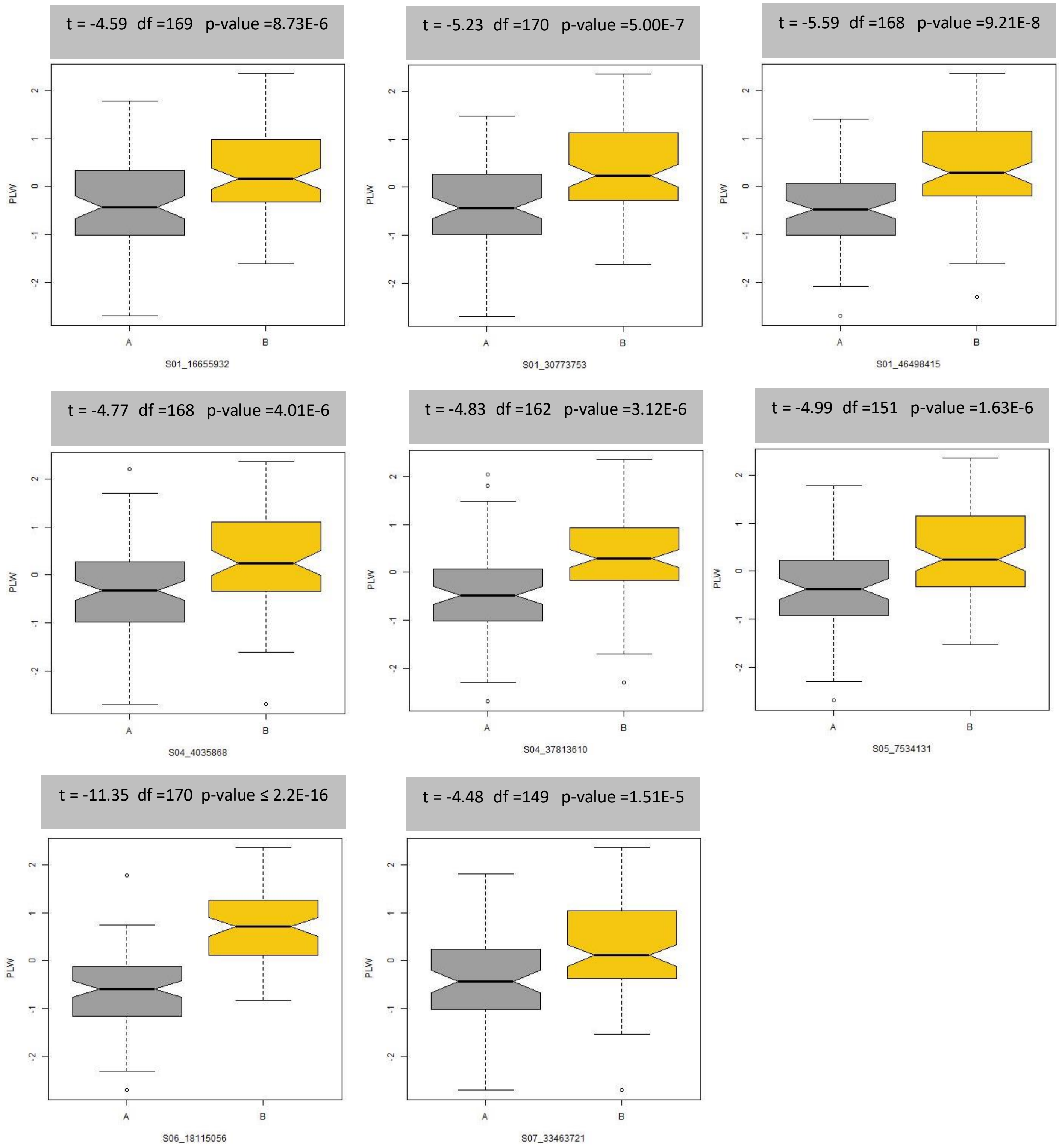
t = -4.59 df =169 p-value =8.73E-6
PLW
A
B
S01_16655932
t = -5.23 df =170 p-value =5.00E-7
PLW
A
B
S01_30773753
t = -5.59 df =168 p-value =9.21E-8
PLW
A
B
S01_46498415
t = -4.77 df =168 p-value =4.01E-6
PLW
A
B
S04_4035868
t = -4.83 df =162 p-value =3.12E-6
PLW
A
B
S04_37813610
t = -4.99 df =151 p-value =1.63E-6
PLW
A
B
S05_7534131
t = -11.35 df =170 p-value ≤ 2.2E-16
PLW
A
B
S06_18115056
t = -4.48 df =149 p-value =1.51E-5
PLW
A
B
S07_33463721

C

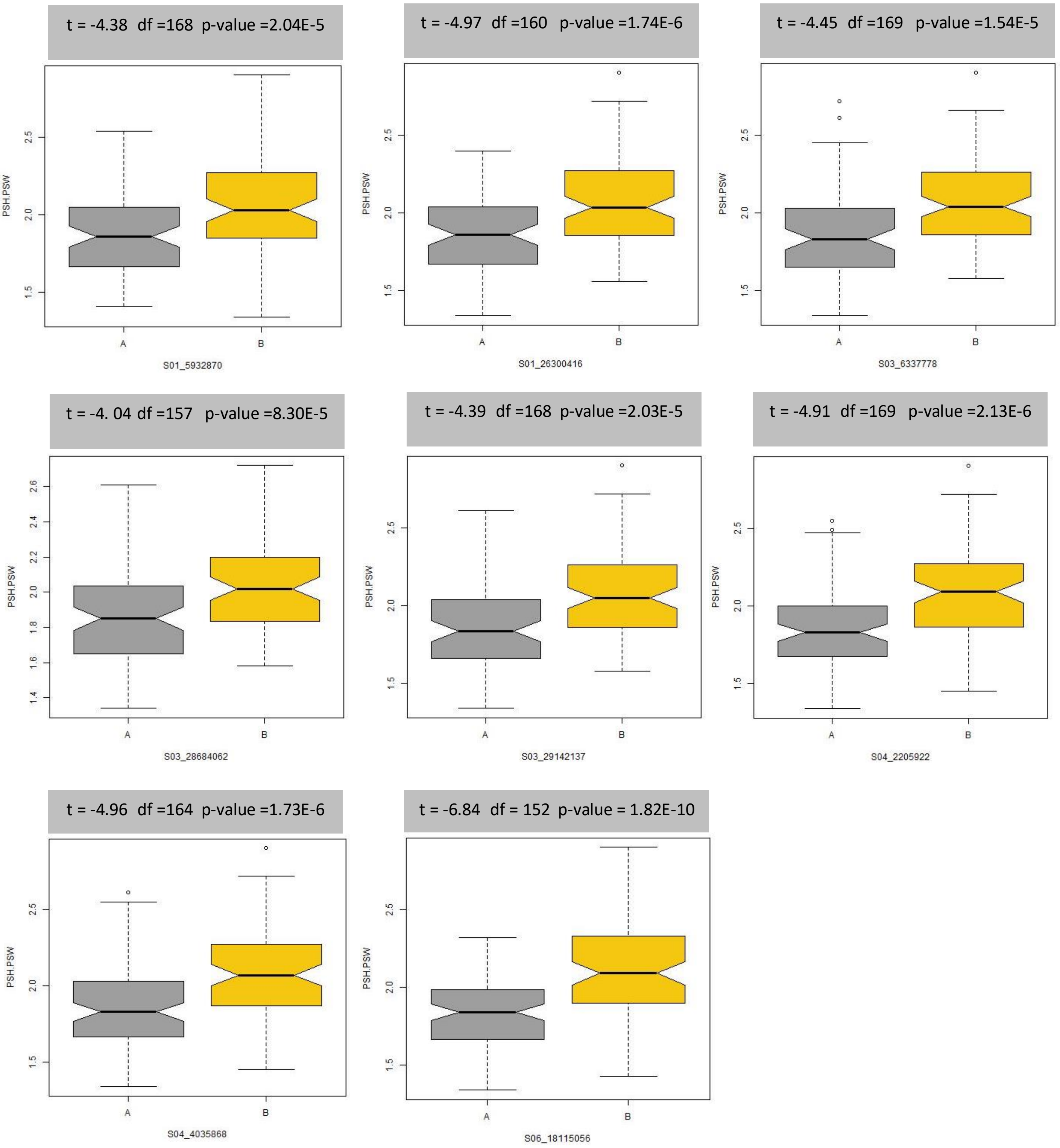
t = -4.38 df =168 p-value =2.04E-5
PSH.PSW
A
B
S01_5932870
t = -4.97 df =160 p-value =1.74E-6
S01_26300416
t = -4.45 df =169 p-value =1.54E-5
S03_6337778
t = -4. 04 df =157 p-value =8.30E-5
S03_28684062
t = -4.39 df =168 p-value =2.03E-5
S03_29142137
t = -4.91 df =169 p-value =2.13E-6
S04_2205922
t = -4.96 df =164 p-value =1.73E-6
S04_4035868
t = -6.84 df = 152 p-value = 1.82E-10
S06_18115056

D

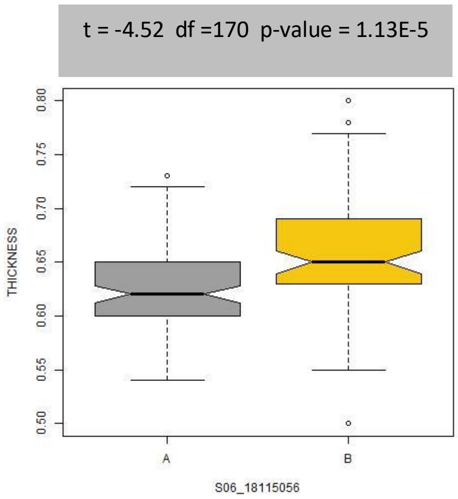
t = -4.52 df =170 p-value = 1.13E-5
THICKNESS
0.50
0.55
0.60
0.65
0.70
0.75
0.80
A
B
S06_18115056

E

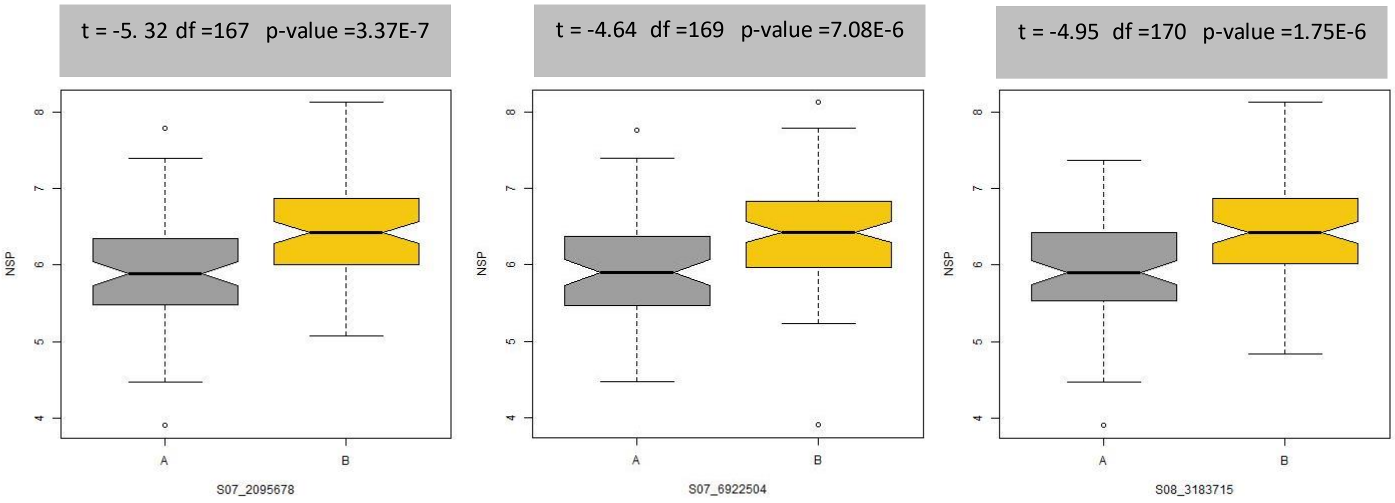
t = -5. 32 df =167 p-value =3.37E-7
t = -4.64 df =169 p-value =7.08E-6
t = -4.95 df =170 p-value =1.75E-6
NSP
4
5
6
7
8
A
B
S07_2095678
S07_6922504
S08_3183715

F

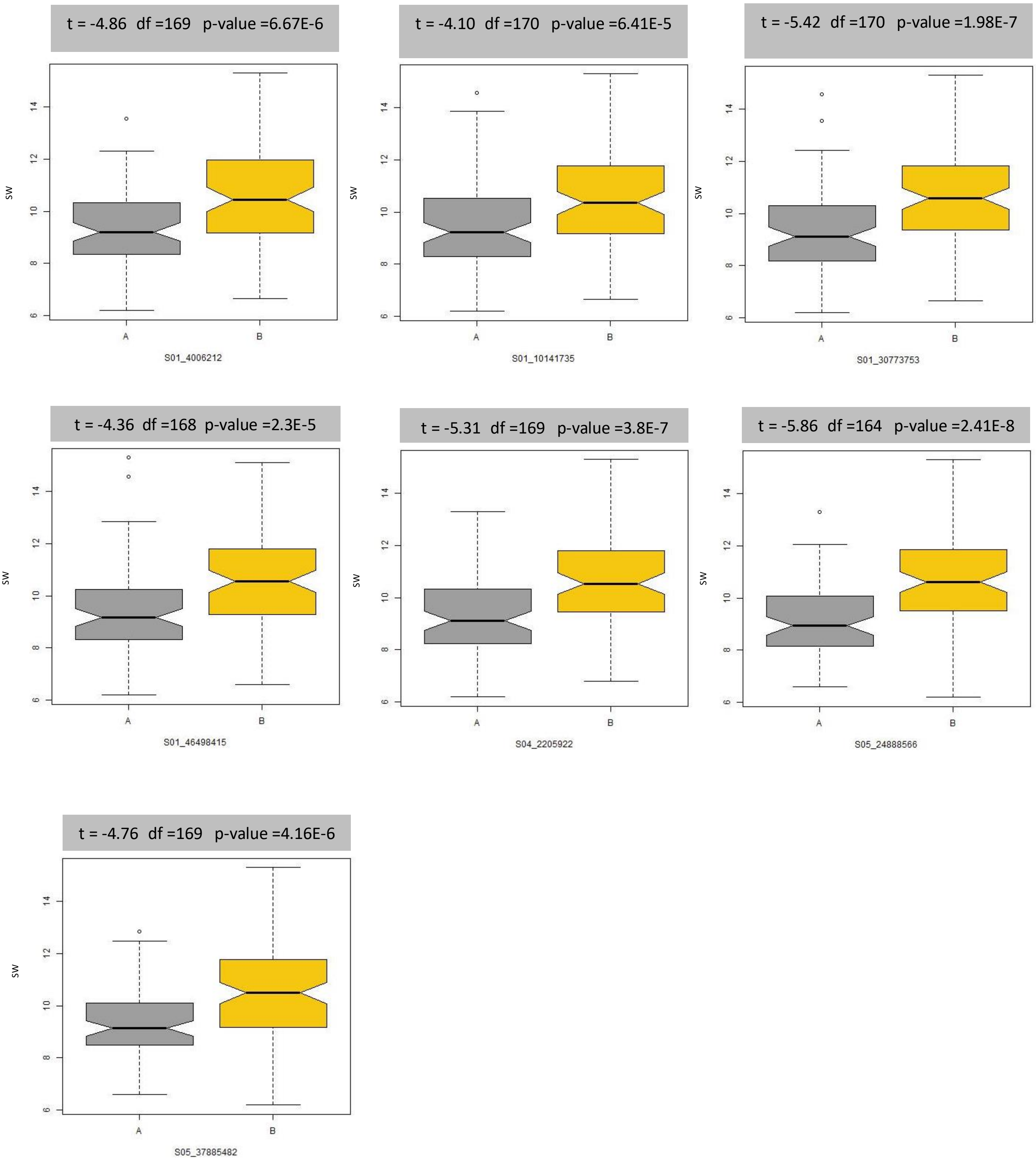
t = -4.86 df =169 p-value =6.67E-6
sw
S01_4006212
t = -4.10 df =170 p-value =6.41E-5
S01_10141735
t = -5.42 df =170 p-value =1.98E-7
S01_30773753
t = -4.36 df =168 p-value =2.3E-5
S01_46498415
t = -5.31 df =169 p-value =3.8E-7
S04_2205922
t = -5.86 df =164 p-value =2.41E-8
S05_24888566
t = -4.76 df =169 p-value =4.16E-6
S05_37885482
A
B
6
8
10
12
14

Figure S6. Barplots showing the probability [-Log10(P)] of the X^2 contingency tests obtained between EPC and each SNP marker included in the genetic map of the TUM RIL population. In each linkage group, markers are represented according to the order obtained in the linkage genetic map. The red line represents a significance level of -Log10 (0.05) after Bonferroni correction (α= 4.226). The asterisk marks represent significant differences after Bonferroni correction at * 0.01 > P < 0.05; ** 0.01 > P < 0.001; ***P< 0.001.

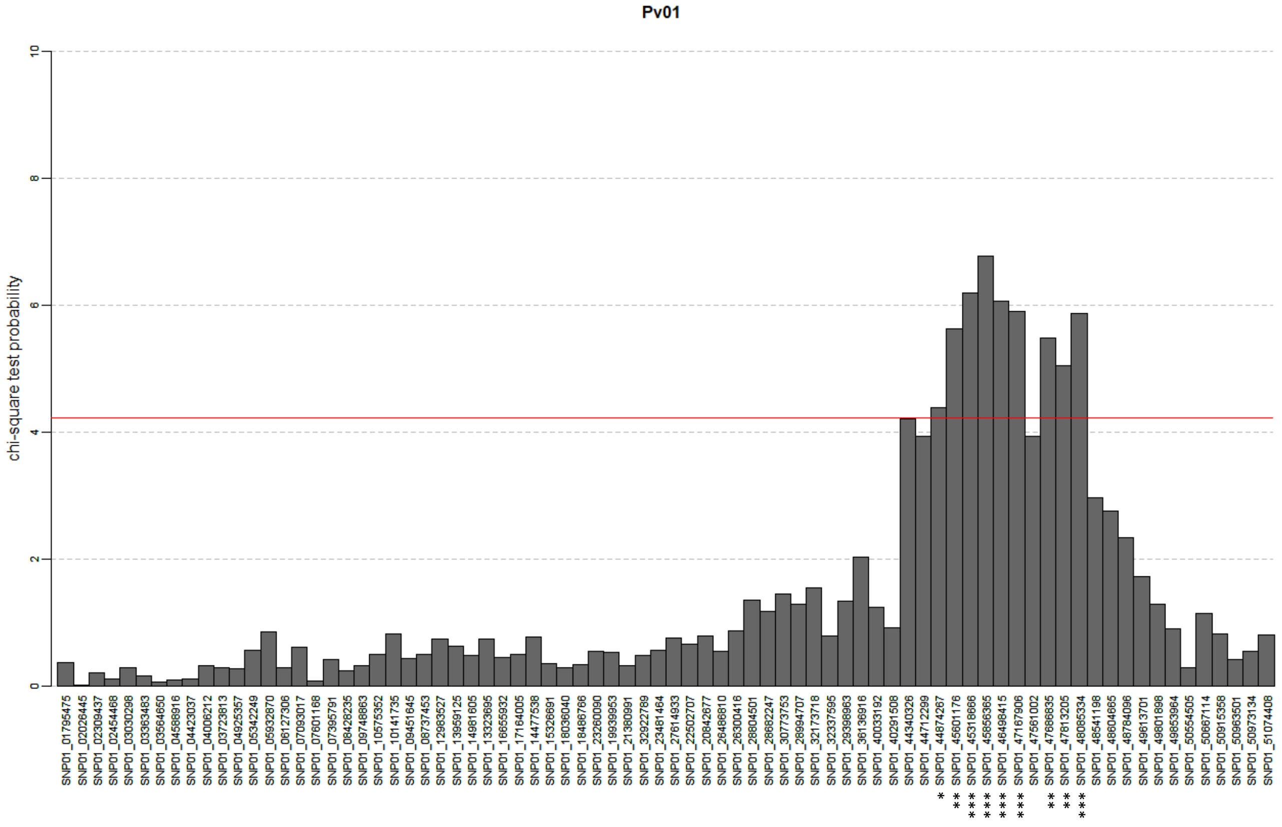

Figure S6. Continues.

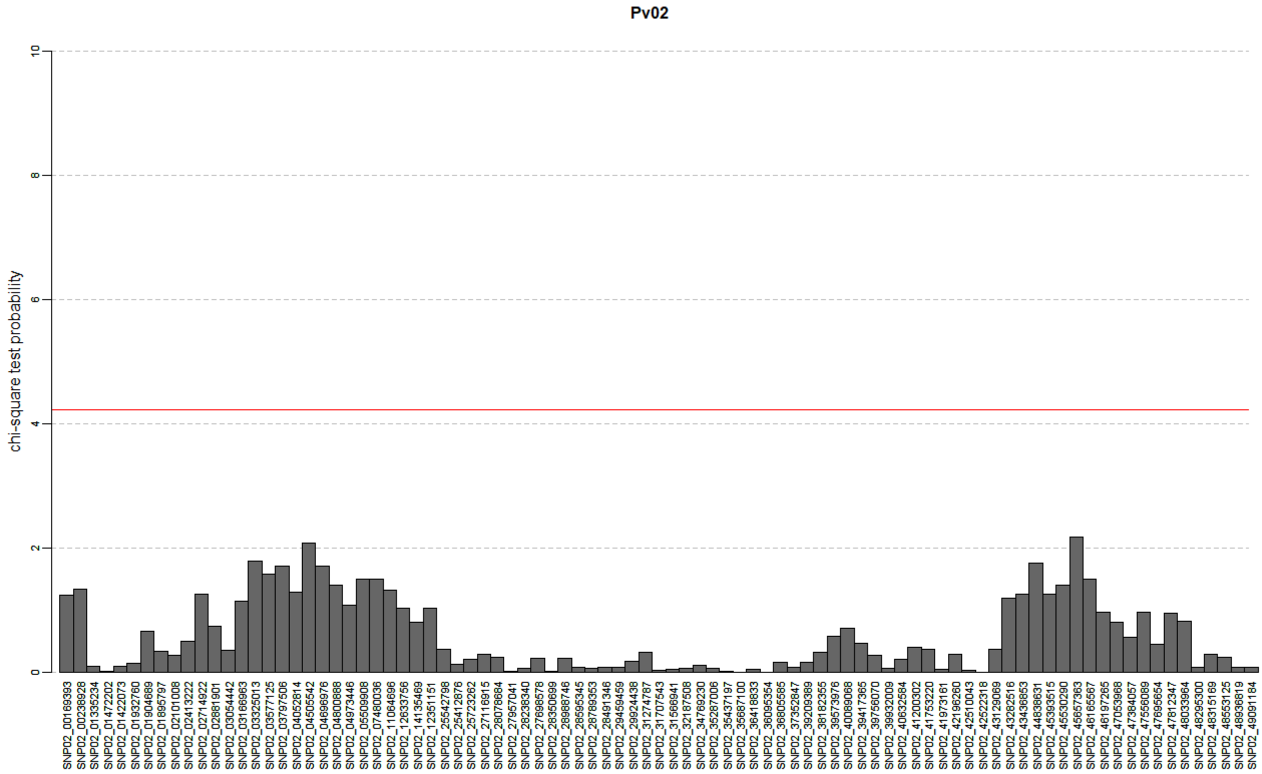

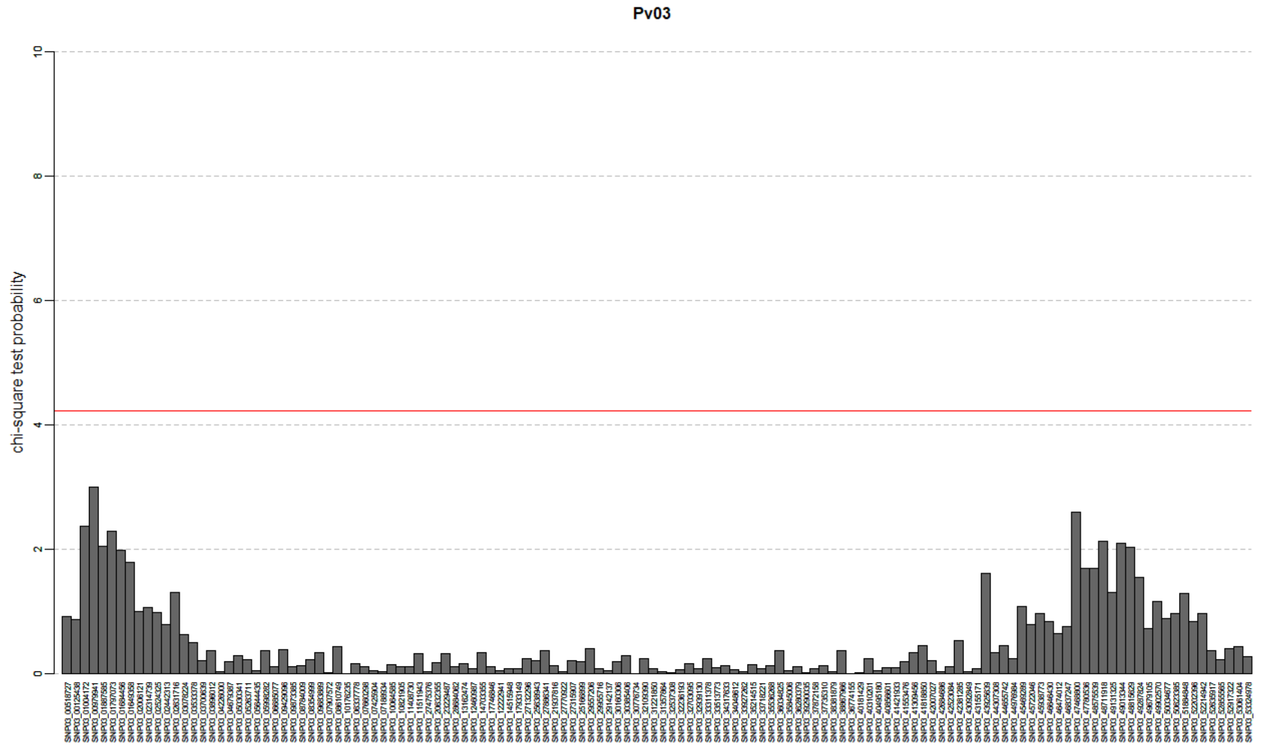

Figure S6. Continues.

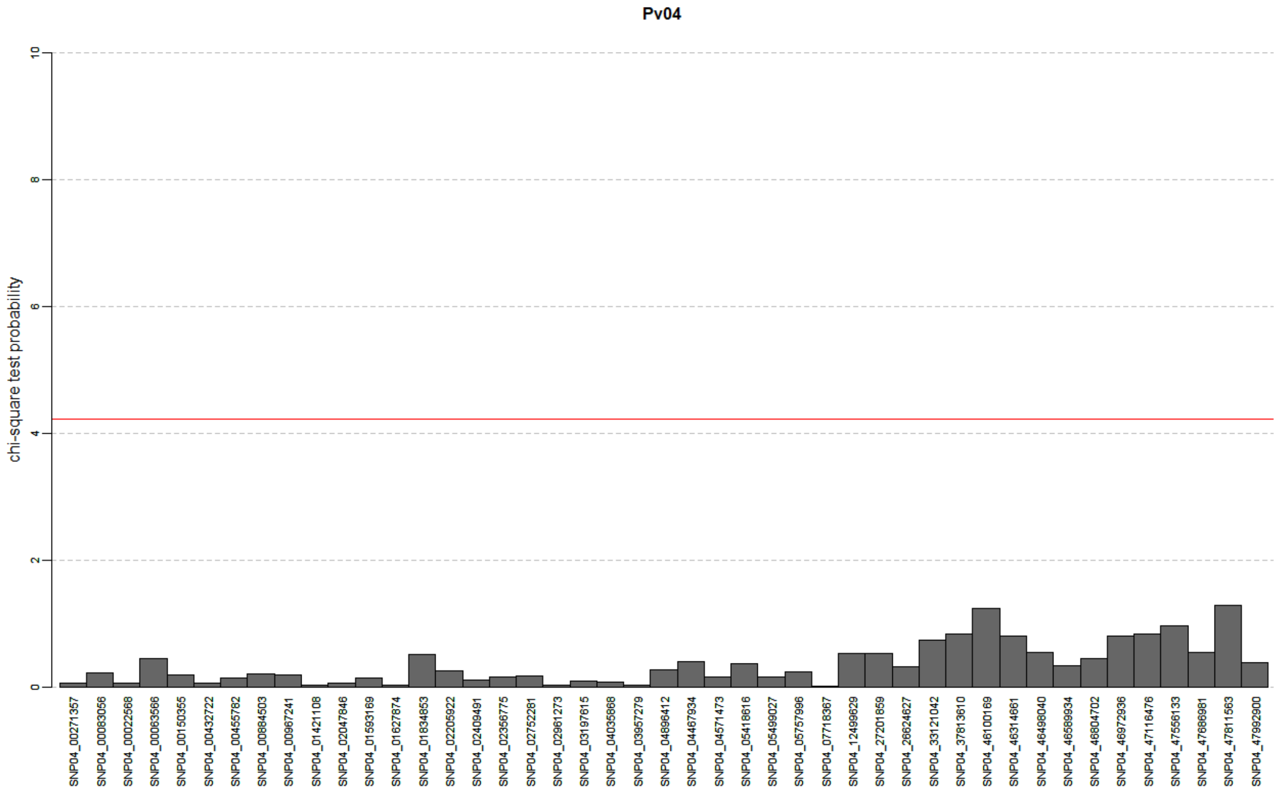

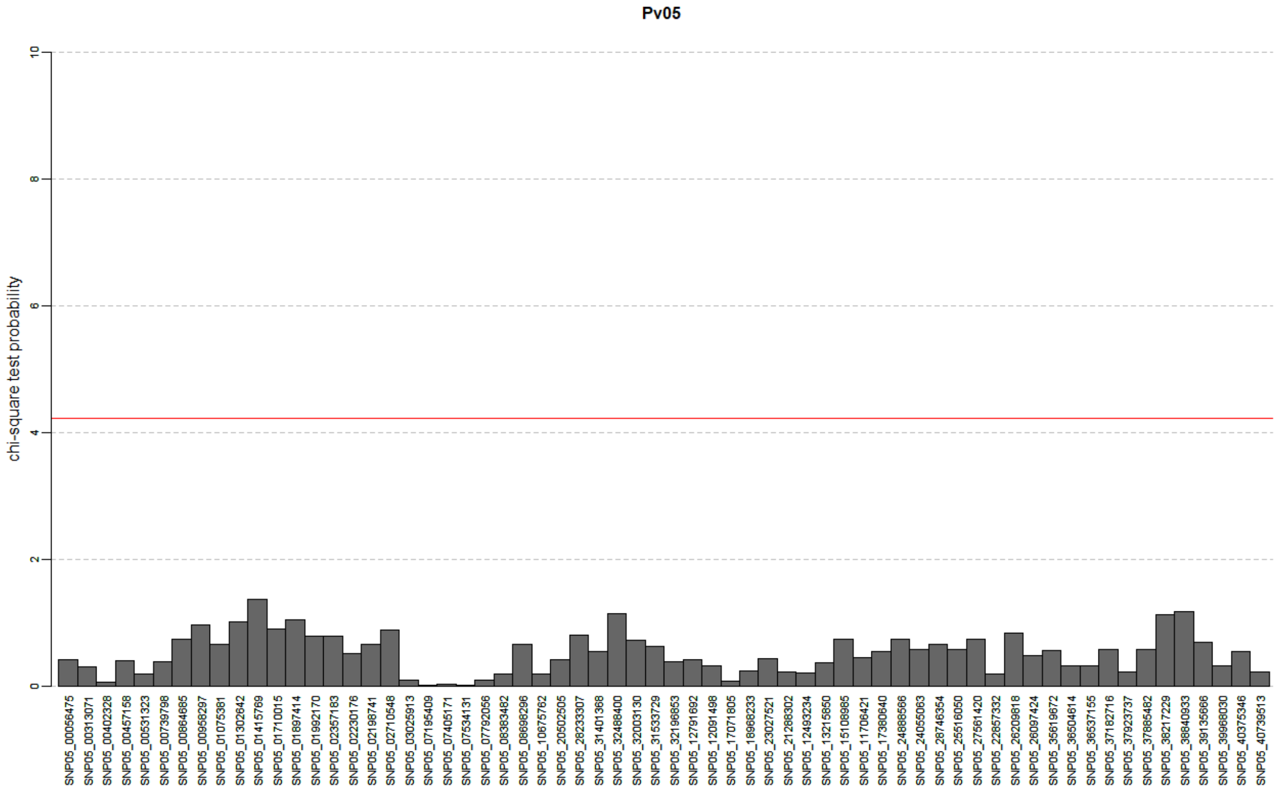

Figure S6. Continues.

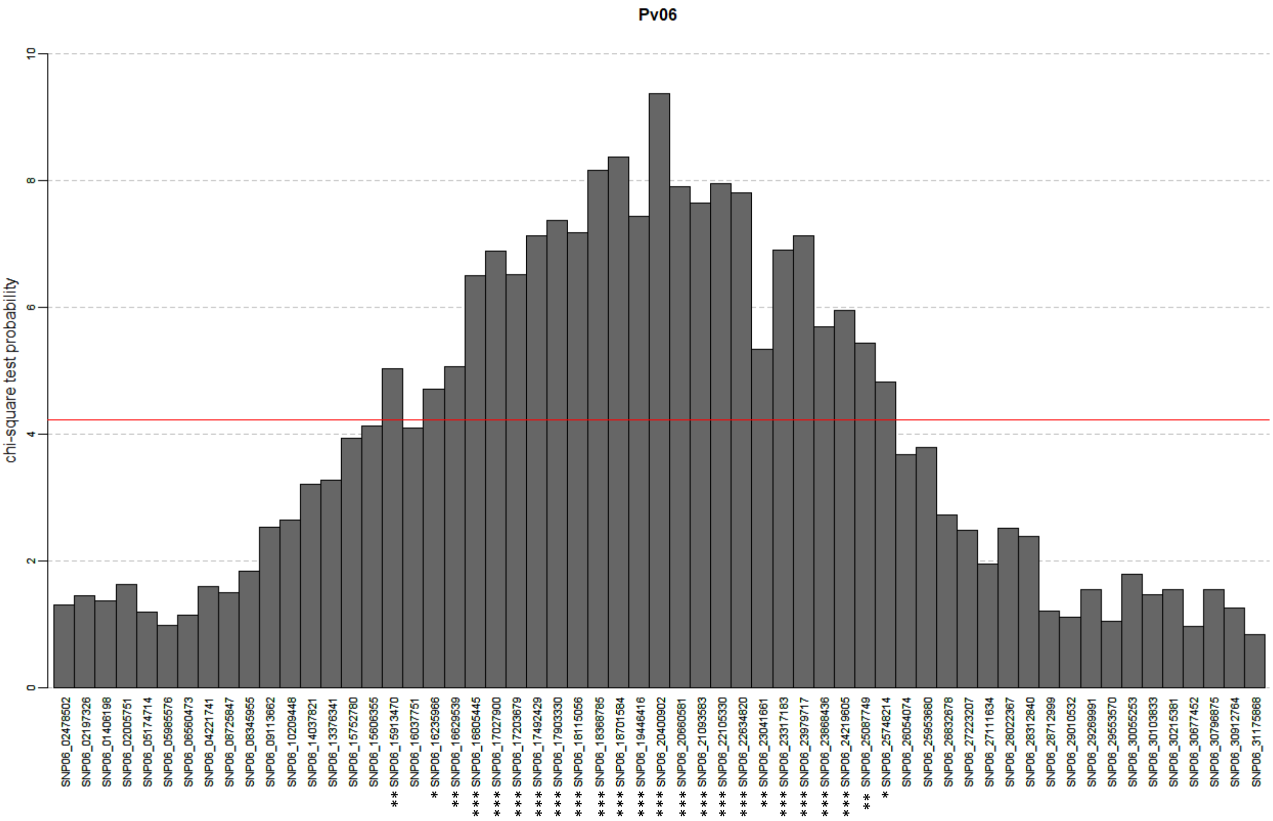

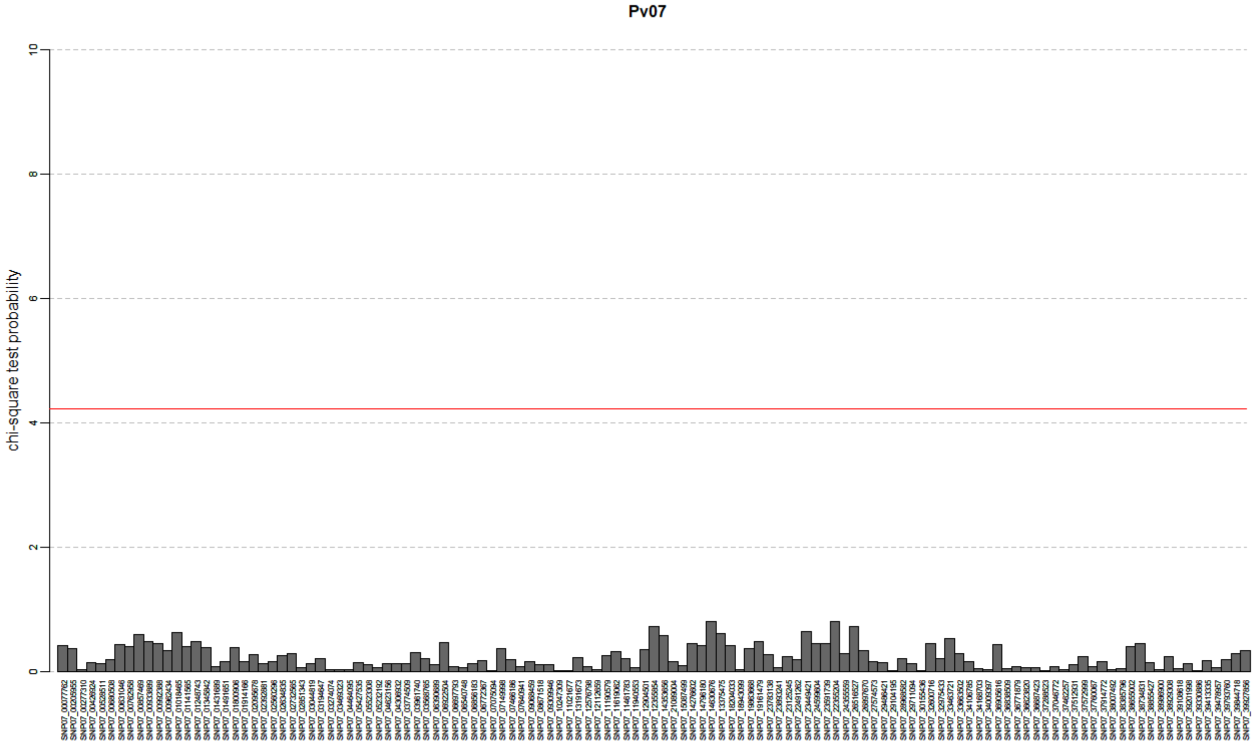

Figure S6. Continues.

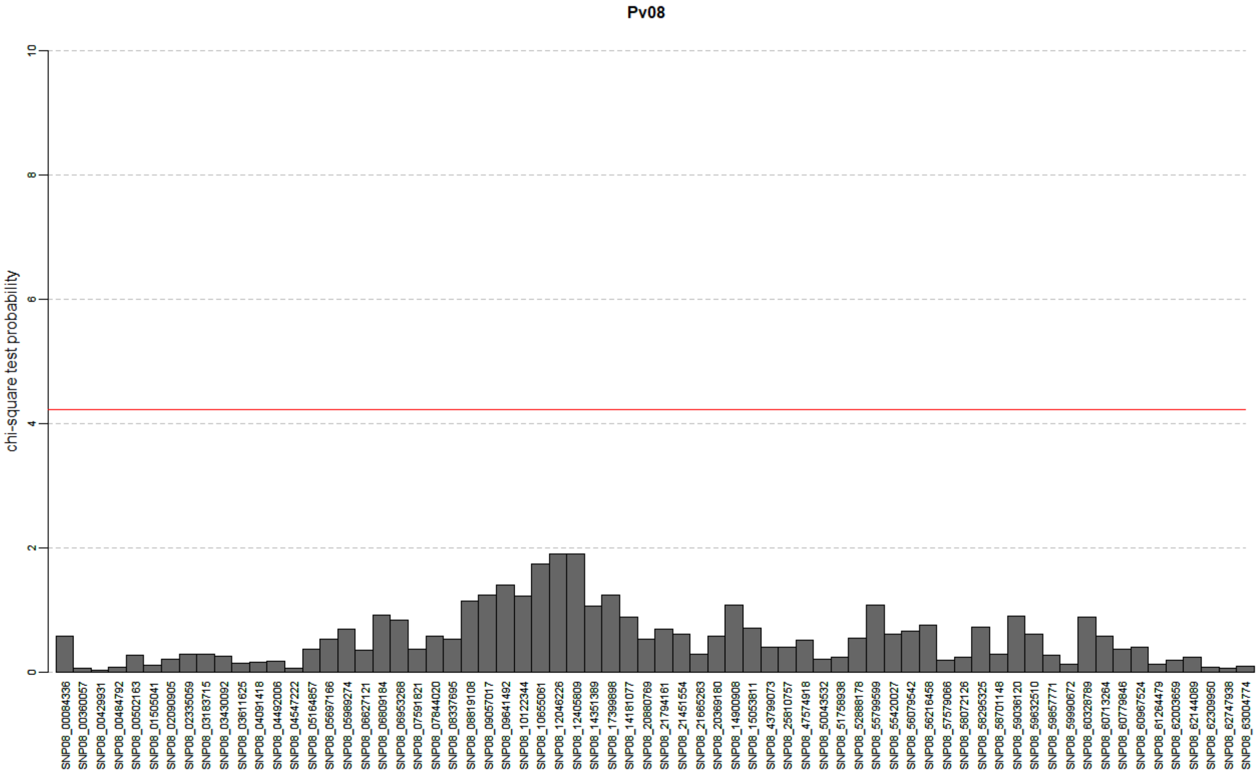

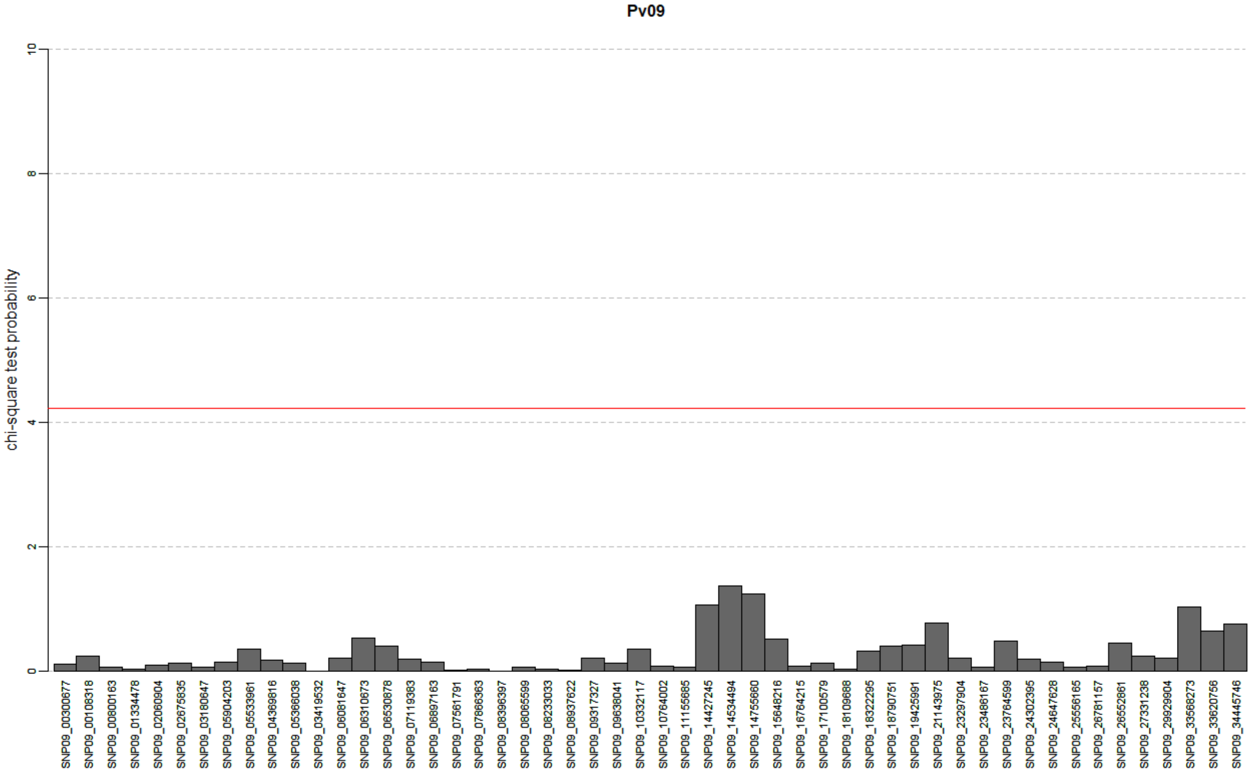

Figure S6. Continues.

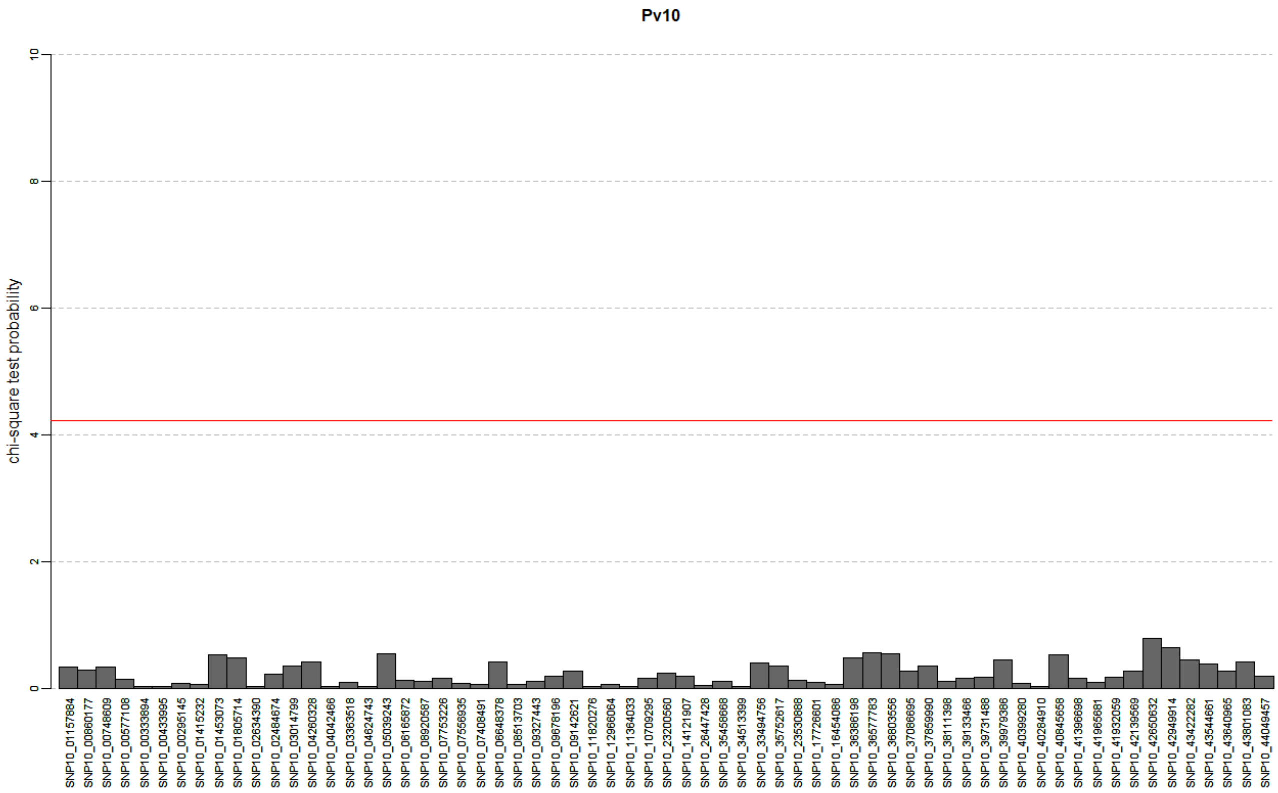

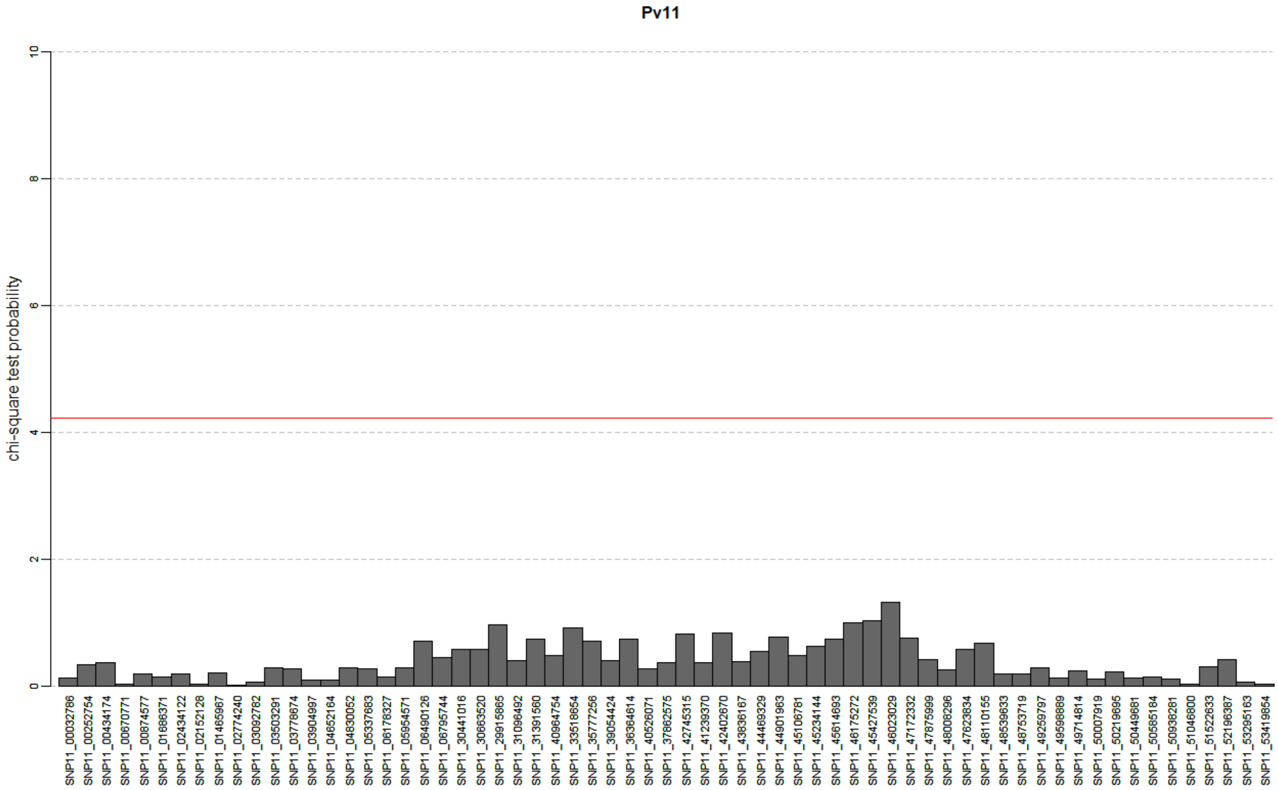

Figure S7. Bar plots of enriched terms for GO_ORA (over-representation analysis). A. Enrichment analysis from genes underlying consensus QTL for PMTs. B . Enrichment analysis from genes underlying regions associated with EPC in a subset of the SDP. Bar plot visualizes gene count as bar height and colored by enrichment scores such as adjusted p values.

A

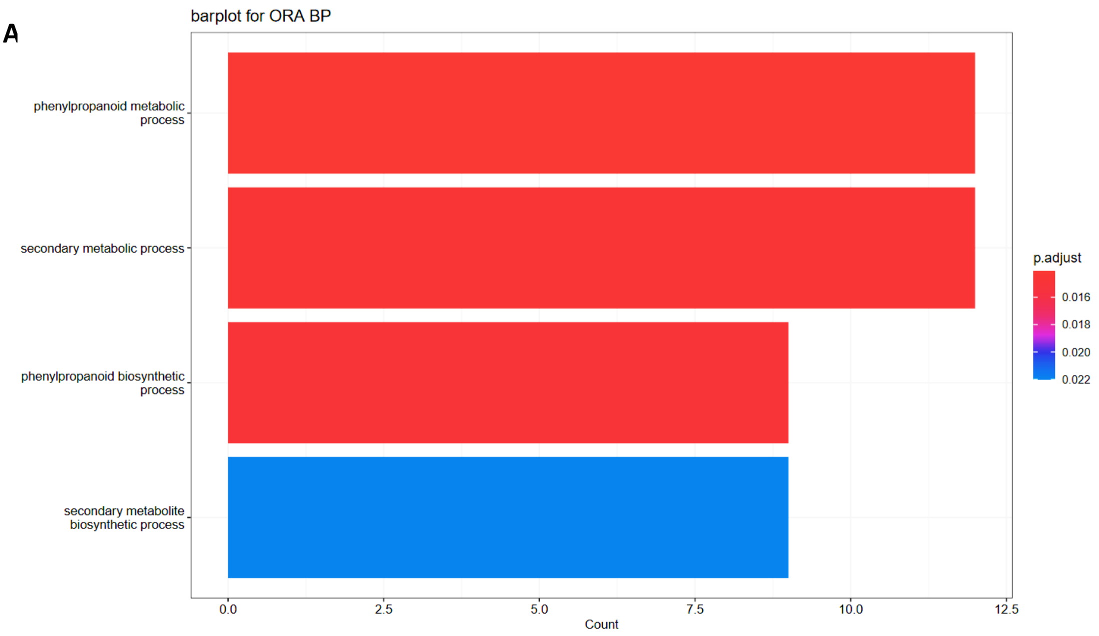

B

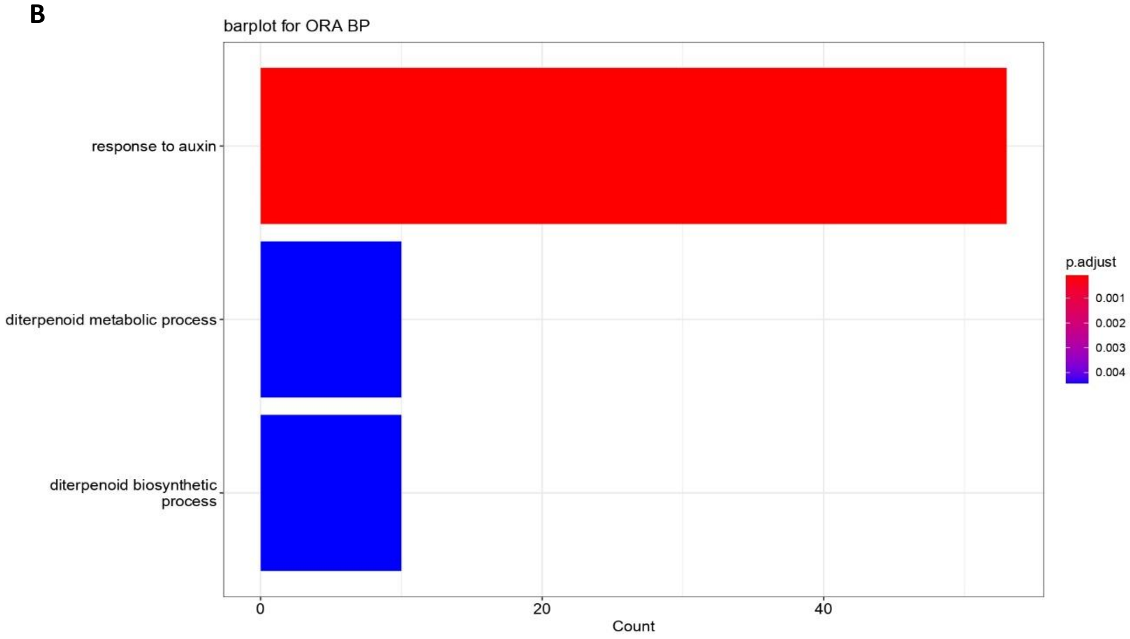

Supplement: Supplementary file 1 — Supplementary file1 (PDF 1891 kb) [file 122_2023_4516_MOESM1_ESM.pdf]
