# Supplementary material for: Genetic control of pod morphological traits and pod edibility in a common bean RIL population
Source: Theor Appl Genet. 2023 Dec 13;137(1):6. doi: 10.1007/s00122-023-04516-6 (PMC10719158; doi:10.1007/s00122-023-04516-6)
Supplement: Supplementary file 2 — Supplementary file2 (PDF 561 kb) [file 122_2023_4516_MOESM2_ESM.pdf]

Table S1. List of accessions included in the selected subset of the Spanish Diversity Panel (Campa et al.2018). GP indicates the gene pool (AN, Andean; MA, Mesoamerican).

Group	SDP Code	Accession/Variety	GP	Type of material
Dry	SDP001	A252	MA	Breeding line
Dry	SDP002	A25 (Andecha)	AN	Old cultivar
Dry	SDP003	A321	MA	Breeding line
Dry	SDP004	A493	MA	Breeding line
Dry	SDP005	AB136	MA	Landrace
Dry	SDP010	BAT93	MA	Breeding line
Dry	SDP035	BGE003121	AN	Landrace
Dry	SDP043	BGE003168	AN	Landrace
Dry	SDP048	BGE003274	AN	Landrace
Dry	SDP051	BGE003298	MA	Landrace
Dry	SDP053	BGE003482	AN	Landrace
Dry	SDP056	BGE003550	AN	Landrace
Dry	SDP057	BGE003554	AN	Landrace
Dry	SDP061	BGE003568	AN	Landrace
Dry	SDP071	BGE004000	AN	Landrace
Dry	SDP080	BGE004452	MA	Landrace
Dry	SDP081	BGE004453	MA	Landrace
Dry	SDP082	BGE004454	MA	Landrace
Dry	SDP083	BGE004459	AN	Landrace
Dry	SDP092	BGE009979	AN	Landrace
Dry	SDP138	BGE023679	AN	Landrace
Dry	SDP141	BGE024699	AN	Landrace
Dry	SDP148	BGE025739	AN	Landrace
Dry	SDP149	BGE025740	MA	Landrace
Dry	SDP150	BGE025745	AN	Landrace
Dry	SDP166	BGE027962	AN	Landrace
Dry	SDP169	BGE028947	AN	Landrace
Dry	SDP173	BGE028964	AN	Landrace
Dry	SDP190	BGE040418	AN	Landrace
Dry	SDP209	BRB130	AN	Breeding line
Dry	SDP212	CN_220	MA	Landrace
Dry	SDP217	CN_227	AN	Landrace
Dry	SDP225	Cornell49242	MA	Breeding line
Dry	SDP227	DOR364	MA	Breeding line
Dry	SDP235	G12508	MA	Landrace
Dry	SDP236	G12910	MA	Landrace
Dry	SDP237	G13467	MA	Landrace
Dry	SDP238	G19833	AN	Landrace
Dry	SDP239	G02333	MA	Landrace
Dry	SDP248	IVT7214	MA	Breeding line
Dry	SDP249	Kaboon	AN	Old cultivar

Group	SDP Code	Accession/Variety	GP	Type of material
Dry	SDP256	MDRK	AN	Old cultivar
Dry	SDP261	Mex222	MA	Old cultivar
Dry	SDP262	Michelite	MA	Old cultivar
Dry	SDP263	N11277	MA	Landrace
Dry	SDP264	N11283	MA	Landrace
Dry	SDP272	PI207262	MA	Landrace
Dry	SDP276	PerryMarrow	AN	Old cultivar
Dry	SDP277	Porrillo Sintetico	MA	Old cultivar
Dry	SDP281	AO-1012-29-3-3A	AN	Breeding line
Dry	SDP282	PR1464-4	MA	Breeding line
Dry	SDP283	PR1464-6	MA	Breeding line
Dry	SDP285	SEL1308	MA	Breeding line
Dry	SDP286	SEL1360	MA	Breeding line
Dry	SDP290	SanilacBc6_Are	MA	Breeding line
Dry	SDP291	Sanilac	MA	Old cultivar
Dry	SDP292	TO	MA	Old cultivar
Dry	SDP294	TU	MA	Old cultivar
Dry	SDP296	V169	AN	Landrace
Dry	SDP297	V203	AN	Landrace
Dry	SDP298	V205	AN	Landrace
Dry	SDP299	V206	AN	Landrace
Dry	SDP301	V208	AN	Landrace
Dry	SDP302	V213	AN	Landrace
Dry	SDP303	V226	MA	Landrace
Dry	SDP306	Widusa	MA	Old cultivar
Dry	SDP307	X2776	AN	Elite cultivar
Dry	SDP308	Xana	AN	Elite cultivar
Snap	SDP006	Amarilla Capitano	AN	Elite cultivar
Snap	SDP007	Amethyst	AN	Elite cultivar
Snap	SDP008	Anellino di trento	AN	Elite cultivar
Snap	SDP009	Astrel	AN	Elite cultivar
Snap	SDP011	Beurre de Rocquencourt	AN	Old cultivar
Snap	SDP049	BGE003283	AN	Landrace
Snap	SDP060	BGE003562	AN	Landrace
Snap	SDP084	BGE004489	AN	Landrace
Snap	SDP114	BGE013981	AN	Landrace
Snap	SDP136	BGE022837	AN	Landrace
Snap	SDP137	BGE023190	AN	Landrace
Snap	SDP192	BGE040527	AN	Landrace
Snap	SDP203	Bilma	MA	Elite cultivar
Snap	SDP204	Bina	AN	Elite cultivar
Snap	SDP205	Blauhilde	MA	Elite cultivar
Snap	SDP206	Bluevetta	AN	Elite cultivar
Snap	SDP207	Boca de Dragon	AN	Elite cultivar

Group	SDP Code	Accession/Variety	GP	Type of material
Snap	SDP208	Borlotto Rosso	AN	Elite cultivar
Snap	SDP210	Brown Ducth	AN	Elite cultivar
Snap	SDP211	Buenos Aires roja	AN	Old cultivar
Snap	SDP223	Cobra	AN	Elite cultivar
Snap	SDP224	Contender	AN	Old cultivar
Snap	SDP226	Donna	MA	Elite cultivar
Snap	SDP228	Dorabel	AN	Elite cultivar
Snap	SDP229	Dublette	AN	Elite cultivar
Snap	SDP230	Duplika	AN	Elite cultivar
Snap	SDP231	Emilia	AN	Elite cultivar
Snap	SDP232	Fin de Bagnols	AN	Old cultivar
Snap	SDP233	Finbel	AN	Elite cultivar
Snap	SDP234	Florencia	MA	Elite cultivar
Snap	SDP240	Garonel	AN	Elite cultivar
Snap	SDP241	Garrafal Enana	AN	Old cultivar
Snap	SDP242	Gloire de Saumur	AN	Old cultivar
Snap	SDP244	Golden Teepee	AN	Elite cultivar
Snap	SDP245	GoldMarie	MA	Elite cultivar
Snap	SDP245	Garrafal Oro	AN	Old cultivar
Snap	SDP246	Helda	MA	Old cultivar
Snap	SDP247	Ilerda	AN	Elite cultivar
Snap	SDP251	Maneca de los Mercados	AN	Elite cultivar
Snap	SDP252	Manteca Rocquencourt	AN	Elite cultivar
Snap	SDP253	Maravilla de Venecia	AN	Old cultivar
Snap	SDP254	Marbel	AN	Elite cultivar
Snap	SDP255	Marconi	MA	Elite cultivar
Snap	SDP257	Midas	AN	Elite cultivar
Snap	SDP258	Monel	AN	Elite cultivar
Snap	SDP259	Musica	MA	Elite cultivar
Snap	SDP260	Meraviglia di Venezia	AN	Elite cultivar
Snap	SDP265	Nassau	AN	Elite cultivar
Snap	SDP266	Neckargold	MA	Elite cultivar
Snap	SDP267	Novirex	AN	Elite cultivar
Snap	SDP268	Nuria	AN	Elite cultivar
Snap	SDP269	Oxinel	AN	Elite cultivar
Snap	SDP270	Patxi	MA	Elite cultivar
Snap	SDP271	Perfeccion Negra Polo	AN	Elite cultivar
Snap	SDP273	Prinzessa	AN	Elite cultivar
Snap	SDP274	Planeta	MA	Elite cultivar
Snap	SDP275	Primel	AN	Elite cultivar
Snap	SDP278	Roma II	AN	Elite cultivar
Snap	SDP279	Rocdor	AN	Elite cultivar
Snap	SDP280	Royalnel	AN	Elite cultivar
Snap	SDP284	Saxa	AN	Elite cultivar

Group	SDP Code	Accession/Variety	GP	Type of material
Snap	SDP287	Slenderette	AN	Elite cultivar
Snap	SDP288	Superba	AN	Elite cultivar
Snap	SDP289	Sacha	MA	Elite cultivar
Snap	SDP293	Triomphe de Facy	AN	Old cultivar
Snap	SDP295	Tendergreen	AN	Old cultivar
Snap	SDP300	V207	AN	Landrace
Snap	SDP304	V381	MA	Landrace
Snap	SDP305	Vitalis	MA	Elite cultivar

Table S2. Information about the studies included in the comparative analysis of QTL for PMTs.

Plant Material			Markers		QTLS		Reference
Population/ Panel	Type	Size	Type	N	Trait	N	
Midas x G12873	RIL	65	RFLPs, isozymes, and seed protein	83	PL, PLW, SW	10	Koinange et al. 1996
PC-50 x XAN-159	RIL	63	AFLPs and RAPDs	*	SW	8	Park et al. 2000
DOR364 x G19833	RIL	86	AFLPs, RAPDs, SSRs and SCARs	236	SW	3	Beebe et al. 2006
Minuette x OSU 5630	RIL	80	RAPDs SSRs, EST, and phenotype markers	200	PL, PLW, SW	8	Davis et al. 2006
Jalo x Small White	RIL	190	SSR	70	PL, SW	31	Souza et al. 2017
Xana x Cornell 49424	RIL	104	AFLPs,SSRs, SCARs,ISSRs, RAPDs, seed proteins, and genes	294	SW	3	Pérez-Vega et al. 2010
Cerinza x G10022	$BC_2F_{2:5}$	138	SSRs	151	SW	9	Blair and Izquierdo 2012
DOR364 x Bat477	RIL	113	AFLPs, RAPDs and SSRs	186	SW	27	Blair et al. 2012
PMB0225 x PHA1037	RIL	185	AFLPs, SSRs, SNPs and morphological marker	194	PL, PLW, PSW	8	Yuste-Lisboa et al. 2014
Turkey Diversity Panel	Diversity panel	66	AFLPs, SSRs and SNPs	418	NSP	2	Nemli et al.2014
PHA0419 x Beluga	RIL	179	SNPs, SSRs	634	PL, PLW, PTH, SW, NSP	17	González et al. 2016
Beluga x PHA0399	RIL	60	SNPs, SSRs	634	PL, PTH, SW, NSP	21	González et al. 2016
OSU5446 x RR6950	RIL	177	SNPs	1,689	PL, PLW	3	Hagerty et al. 2016
SER48 x Merlot		76					
SER94 x Merlot	RIL	48	SNPs	666	SW	2	Hoyos-Villegas et al. 2016
SER95 x Merlot		36					
Middle American Diversity Panel	Diversity panel	280	SNPs (GWAS)	>150,000	SW	4	Moghaddam et al. 2016
BK-004-001 x h68-4	RIL	114	SNPs	611	SW	16	Sandhu et al. 2018
CAL96 x MLB-49-89A	RIL	121	SNPs	822	SW	3	Wang et al. 2018
Solwezi x AO-1012-29-3-3A	RIL	210	SNPs	760	SW	4	Kamfwa et al. 2018
Diversity panel	Diversity panel	363	SNPs (GWAS)	84,416	SW	14	Wen et al. 2019

Plant Material			Markers		QTLS		Reference
Population/ Panel	Type	Size	Type	N	Trait	N	
Goli x AND1007	RIL	100	SNPs	240	SW, NSP	15	Geravandi et al. 2020
Chinese common bean Panel	Diversity panel	3,095	SSRs	116	SW	4	Lei et al. 2020
Xana x BAT93	RIL	145	SNPs	497	PL, PLW, SW, NSP	22	Murube et al. 2020
Xana x Cornell 49424	RIL	115	SNPs, Indels, SSRs, SCARs,STSs, RAPD and seed proteins	762	PL, PLW, PTH, SW, NSP	21	Murube et al. 2020
Tiber x Starezagerski čern	RIL	82	AFLPS and SSRs	123	SW	7	Sedlar et al. 2020
Spanish Diversity Panel	Diversity panel	301	SNPs (GWAS)	346,819	PL, PLW, PSH/PSW, NSP	25	García-Fernández et al. 2021a

*Information not found

PL: pod length; PLW: pod width; PSH/PSW: fit of the cross-section to circularity; PTH: pod thickness; NSP: number of seeds per pod; SW: seed weight.

Table S3. List of QTL detected for PMTs in the TUM population organized by trait that colocalize with QTL previously reported.

Trait	QTL ID[1]	QTL ID[2]	Chr	Flanking markers	Start	End	Reference
PL	PL1.4^{TUM}	PL1.2^{GA}	Pv01	ss715639829-ss715639588	18,460,106	27,389,335	Geravandi et al. 2020
		PL1.1^{XB}	Pv01	SNP01_288-SNP01_295	29,932,212	33,789,966	Murube et al. 2020
		PL1^{PP}	Pv01	BMc324-BM200	32,413,315	48,348,176	Yuste-Lisboa et al. 2014
	PL1.5^{TUM}	PL-1MA ePL-1MA	Pv01	FIN-BMC224	44,856,086	47,409,645	González et al. 2016
		PodL01_45.8	Pv01	45582871-45878761	45,582,871	45,878,761	García-Fernández et al. 2021a
		PodLCol01_48	Pv01	48090873-48454962	48,090,873	48,454,962	García-Fernández et al. 2021a
	PL4.1^{TUM}	PL4.1^{GA}	Pv04	ss715648827	15,105,934	15,405,934	Geravandi et al. 2020
		PL4^{PP}	Pv04	BM140-E45M38-216	12,733,142	41,879,480	Yuste-Lisboa et al. 2014
	PL6.1^{TUM}	ePL-6MA	Pv06	BMC238-IAC047	7,858,085	21,009,895	González et al. 2016
		BM187	Pv06	19569705-19569900	19,569,705	19,569,900	Souza et al.2017
		Pod_height	Pv06	SS715648561-BM187	13,324,729	19,569,900	Davis et al. 2014
PLW	PLW1.2^{TUM} PLW1.3^{TUM}	PWI1^{PP}	Pv01	BMc324-BM200	32413315	48,348,176	Yuste-Lisboa et al. 2014
	PLW6.1^{TUM}	NSPLS06_18.4	Pv06	18457867-19126326	18,457,867	19,126,326	García-Fernández et al. 2021a
		PWI6^{XB}	Pv06	SNP06_200-SNP06_247	18,368,762	18,781,236	Murube et al. 2020
		PodWidth	Pv06	ss715650936-ss715647379	12,098,258	22,279,779	Davis et al. 2014
PSH/PSW	PSH/PSW6.1^{TUM}	NSPLS06_18.4	Pv06	18457867-19126326	18,457,867	19,126,326	García-Fernández et al. 2021a
NSP	NSP7.2^{TUM}	NSP7^{XC}	Pv07	SNP_195-SNP_2278	6,534,445	7,107,066	Murube et al. 2020
SW	SW1.3^{TUM}	SW-1MA	Pv01	IAC076-IAC089	26,506,660	30,514,857	González et al. 2016
	SW4.1^{TUM}	SW4.1^{GA}	Pv04	ss715647804	2,087,956		Geravandi et al. 2020
	SW5.2^{TUM}	Sw5.3	Pv05	BM155–BM175	37,077,612	38,717,854	Blair and Izquierdo 2012
		SW-5MA	Pv05	BM175-BMD020	37,077,612	38,501,256	González et al. 2016

PL: pod length; PLW: pod width; PSH/PSW: fit of the cross-section to circularity; PTH: pod thickness; NSP: number of seeds per pod; SW: seed weight.

(1) QTL detected in the TUM RIL population; (2) QTL detected in other studies.

Table S4. t-Test table for equality of means regarding the PMTs of the edible and nonedible phenotype class in the TUM population.

Pod trait	EPC	t-test		
		Means	t-value	P-value
PL	Nonedible	14.796	-2.097	*
	Edible	15.693		
PLW	Nonedible	1.217	-3.9719	***
	Edible	1.378		
PTH	Nonedible	0.636	-1.837	ns
	Edible	0.651		
PSH/PSW	Nonedible	1.934	-2.485	*
	Edible	2.069		
NSP	Nonedible	6.182	0.002	ns
	Edible	6.182		
SW	Nonedible	9.927	-1.491	ns
	Edible	10.475		

$\alpha > 0.05$ ns (not significant); * $0.01 > \alpha < 0.05$; ** $0.01 > \alpha < 0.001$; ***< 0.001

Table S5. List of SNPs showing significant [-LOG10(p) > 3] association with EPC ('snap' vs. 'non-snap') identified by SL-GWAS method (MLM) in a subset of the SDP. MAF: minor allele frequency; df: degrees of freedom. (Excel file)

Table S6. List of genes underlying bean consensus QTL for PMTs detected in this study. Genes located at overlapping positions with EPC regions detected in a subset of the SDP and in the TUM RIL population are also indicated. (Excel file)

Table S7. Genes identified with functional annotation associated with the phenylpropanoid secondary metabolite process detected by GO ontology enrichment analysis in the consensus QTL associated with PMTs.

Consensus QTL ID	Gene ID	Functional Annotation	Size	Chromosome	Start	End
Pod6.2^{pv}	Phvul.006g074000	(1 Of 2) Pthr11709:Sf68 - Laccase-13-Related	2987	Chr06	18653069	18656055
Pod1.5^{pv}	Phvul.001g122100	(1 Of 7) Pthr11709:Sf103 - Laccase-16	1006	Chr01	33803162	33804167
Pod1.6^{pv}	Phvul.001g145000	(1 Of 10) Pthr21495:Sf71 - Dirigent Protein 1-Related	579	Chr01	38968769	38969347
Pod1.6^{pv}	Phvul.001g145100	(1 Of 10) Pthr21495:Sf71 - Dirigent Protein 1-Related	795	Chr01	38976134	38976928
Pod1.6^{pv}	Phvul.001g145200	(1 Of 10) Pthr21495:Sf71 - Dirigent Protein 1-Related	579	Chr01	38985654	38986232
Pod1.6^{pv}	Phvul.001g145300	(1 Of 10) Pthr21495:Sf71 - Dirigent Protein 1-Related	1259	Chr01	39008950	39010208
Pod1.6^{pv}	Phvul.001g145500	(1 Of 10) Pthr21495:Sf71 - Dirigent Protein 1-Related	558	Chr01	39062907	39063464
Pod1.6^{pv}	Phvul.001g145600	(1 Of 10) Pthr21495:Sf71 - Dirigent Protein 1-Related	990	Chr01	39112918	39113907
Pod1.6^{pv}	Phvul.001g145700	(1 Of 10) Pthr21495:Sf71 - Dirigent Protein 1-Related	900	Chr01	39197886	39198785
Pod1.6^{pv}	Phvul.001g145800	(1 Of 10) Pthr21495:Sf71 - Dirigent Protein 1-Related	1014	Chr01	39210683	39211696
Pod1.6^{pv}	Phvul.001g145900	(1 Of 10) Pthr21495:Sf71 - Dirigent Protein 1-Related	648	Chr01	39230611	39231258
Pod1.6^{pv}	Phvul.001g148600	(1 Of 10) Pthr21495:Sf71 - Dirigent Protein 1-Related	9441	Chr01	39550592	39560032

Table S8. List of genes underlying genomic regions associated with EPC ('snap' vs. 'non-snap') in a subset of the SDP. (Excel file)

Table S9. Genes with functional annotation associated with the auxin response detected by GO ontology enrichment analysis in the genomic regions associated with EPC ('snap' vs. 'non-snap') on a subset of the SDP. Auxin response genes underlying the genomic regions involved in the control of EPC in the TUM RIL population are also indicated. (Excel file)

Table S10. Genes identified with functional annotation associated with the diterpenoid metabolic and biosynthetic process by GO ontology enrichment analysis in the genomic regions associated with EPC ('snap' vs. 'non-snap') on a subset of the SDP.

QTL ID	Gene ID	Functional annotation	Size	Chromosome	Start	End
Edibility11.3^{SDP}	Phvul.011G107200	(1 of 3) 4.2.3.106 - (E)-beta-ocimene synthase / Beta-ocimene synthase	4669	Chr11	12546726	12551394
Edibility11.3^{SDP}	Phvul.011G142500	(1 of 8) K15803 - (-)-germacrene D synthase (GERD)	4911	Chr11	36608124	36613034
Edibility11.3^{SDP}	Phvul.011G142700	(1 of 8) K15803 - (-)-germacrene D synthase (GERD)	4438	Chr11	36848151	36852588
Edibility11.3^{SDP}	Phvul.011G143100	(1 of 8) K15803 - (-)-germacrene D synthase (GERD)	5295	Chr11	37151836	37157130
Edibility11.3^{SDP}	Phvul.011G143200	(1 of 8) K15803 - (-)-germacrene D synthase (GERD)	4948	Chr11	40679144	40684091
Edibility11.3^{SDP}	Phvul.011G143300	(1 of 14) 4.2.3.22 - Germacradienol synthase / Germacradienol/germacrene-D synthase	5024	Chr11	40605067	40610090
Edibility11.3^{SDP}	Phvul.011G143500	(1 of 8) K15803 - (-)-germacrene D synthase (GERD)	11355	Chr11	40472384	40483738
Edibility11.3^{SDP}	Phvul.011G143600	(1 of 8) K15803 - (-)-germacrene D synthase (GERD)	5078	Chr11	41085383	41090460
Edibility11.3^{SDP}	Phvul.011G143700	(1 of 8) K15803 - (-)-germacrene D synthase (GERD)	7061	Chr11	39774928	39781988
Edibility11.3^{SDP}	Phvul.011G144200	(1 of 14) 4.2.3.22 - Germacradienol synthase / Germacradienol/germacrene-D synthase	5473	Chr11	40149233	40154705
